# Inferring pregnancy episodes and outcomes within a network of observational databases

**DOI:** 10.1371/journal.pone.0192033

**Published:** 2018-02-01

**Authors:** Amy Matcho, Patrick Ryan, Daniel Fife, Dina Gifkins, Chris Knoll, Andrew Friedman

**Affiliations:** 1 Epidemiology Analytics, Janssen Research and Development, LLC, Raritan, New Jersey, United States of America; 2 Epidemiology, Janssen Research and Development, LLC, Titusville, New Jersey, United States of America; 3 Global Labeling, Janssen Research and Development, LLC, Titusville, New Jersey, United States of America; King’s College London, UNITED KINGDOM

## Abstract

Administrative claims and electronic health records are valuable resources for evaluating pharmaceutical effects during pregnancy. However, direct measures of gestational age are generally not available. Establishing a reliable approach to infer the duration and outcome of a pregnancy could improve pharmacovigilance activities. We developed and applied an algorithm to define pregnancy episodes in four observational databases: three US-based claims databases: Truven MarketScan^®^ Commercial Claims and Encounters (CCAE), Truven MarketScan^®^ Multi-state Medicaid (MDCD), and the Optum ClinFormatics^®^ (Optum) database and one non-US database, the United Kingdom (UK) based Clinical Practice Research Datalink (CPRD). Pregnancy outcomes were classified as live births, stillbirths, abortions and ectopic pregnancies. Start dates were estimated using a derived hierarchy of available pregnancy markers, including records such as last menstrual period and nuchal ultrasound dates. Validation included clinical adjudication of 700 electronic Optum and CPRD pregnancy episode profiles to assess the operating characteristics of the algorithm, and a comparison of the algorithm’s Optum pregnancy start estimates to starts based on dates of assisted conception procedures. Distributions of pregnancy outcome types were similar across all four data sources and pregnancy episode lengths found were as expected for all outcomes, excepting term lengths in episodes that used amenorrhea and urine pregnancy tests for start estimation. Validation survey results found highest agreement between reviewer chosen and algorithm operating characteristics for questions assessing pregnancy status and accuracy of outcome category with 99–100% agreement for Optum and CPRD. Outcome date agreement within seven days in either direction ranged from 95–100%, while start date agreement within seven days in either direction ranged from 90–97%. In Optum validation sensitivity analysis, a total of 73% of algorithm estimated starts for live births were in agreement with fertility procedure estimated starts within two weeks in either direction; ectopic pregnancy 77%, stillbirth 47%, and abortion 36%. An algorithm to infer live birth and ectopic pregnancy episodes and outcomes can be applied to multiple observational databases with acceptable accuracy for further epidemiologic research. Less accuracy was found for start date estimations in stillbirth and abortion outcomes in our sensitivity analysis, which may be expected given the nature of the outcomes.

## Introduction

Administrative claims databases and electronic health records are valuable resources for evaluating effects of exposures, including prescription drug exposures during pregnancy, on pregnancy outcomes. Comprehensive drug exposure safety information for pregnant populations is generally unavailable when a drug first comes to market as randomized clinical trials routinely exclude pregnant women from study. Pregnancy registries, usually established when a medication is marketed, are expensive to execute. They capture relatively modest numbers of pregnancies compared to administrative claims databases and electronic health records and are subject to recall bias, especially if the exposure data are collected after delivery.

The use of claims and electronic health record data in the study of pregnancy exposures has several advantages including capture of prescription dispensing data, longitudinal follow-up from drug exposure through pregnancy outcome, potential mother-infant linkage, and large enough samples to study rare pregnancy outcomes. Accurate prescription dispensing, pregnancy episode start dates, and pregnancy episode end dates are necessary to avoid drug exposure misclassification. However, direct measures of gestational age are usually not available in claims databases and electronic health records. This creates challenges when estimating pregnancy start dates and understanding the potential impact of drug exposure on a fetus at a given gestational age.

Prior attempts have been made to infer pregnancy episodes in observational data [1–10]. A few focused exclusively on live births, excluding other birth outcomes such as abortions (6, 7, 10). Some estimated pregnancy start by subtracting an estimated gestational age from the pregnancy outcome date [2, 6, 9, 10], while others used early pregnancy markers or combined both methods to infer start [1, 3–5, 7, 8]. Early pregnancy markers such as the nuchal ultrasound procedure can be used to infer pregnancy start based on clinical care guidelines (recommended gestational age for procedure can be subtracted from procedure date to infer a pregnancy start). A few efforts assigned start of pregnancy to first pregnancy care record instead of inferred gestational age of infant [3, 4, 8]. This approach would generally estimate a later pregnancy start as clinical care is not sought until a few weeks after pregnancy begins. All methods of estimating gestational age have some margin of error. Relying on only one method runs the risks of inaccuracies of that particular method. For example, use of last menstrual period (LMP) could be biased because it is not accurately remembered by the pregnant woman or is not recorded. Because some proxies are more accurate than others, a hierarchical approach that utilizes the collective predictive accuracy of each method could be beneficial when one has multiple sources to estimate gestational age. Also, it is unclear if any of the above algorithms could be applied to other data sources, as none of the prior published work evaluated performance across multiple disparate databases. Therefore, it is advantageous to develop a single validated algorithm against a common data model that can be applied to multiple data sources so exposures and outcomes within different populations can be explored simultaneously. A better case for generalizability of study results can also be made when multiple populations are included in an analysis.

The objective of this study was to create a generic algorithm that could infer pregnancy episodes and a variety of outcomes (including live births, stillbirths, abortions and ectopic pregnancies) across several disparate observational data sources. We sought to incorporate all appropriate pregnancy clinical care records into the pregnancy episode start estimation, rather than simply subtracting an estimated gestational age from the pregnancy outcome date. The algorithm was validated in three US-based claims databases and one UK primary care database. The algorithm was developed in the Observational Medical Outcomes Partnership (OMOP) Common Data Model [11] and could be applied to any observational data source which has been converted to the above data model. It is publicly available at https://github.com/OHDSI/PhenotypeLibrary for use by any organization using this open-source community standard.

## Methods

### Pregnancy episode algorithm details

#### Data sources

We applied the algorithm to four observational, de-identified data sources (descriptions in Table 1).

**Table 1:** Observational data source descriptions.

Database	Category	Number of patient lives (millions)	Dates represented	Additional details
Truven MarketScan^®^ Commercial Claims and Encounters (CCAE)	Employer-based US administrative health claims	~119	1/1/2000-12/31/2013	CCAE represents data from individuals enrolled in US employer-sponsored insurance health plans, including fee-for-service, preferred provider organizations, and capitated health plans. The data includes adjudicated health insurance claims (inpatient, outpatient, and outpatient pharmacy) as well as enrollment data. Additionally, laboratory tests are captured for a subset of the covered lives. Source data dictionaries include National Drug Codes (NDC) for prescriptions, CPT-4, HCPCs, ICD-9-CM for procedures, ICD-9-CM for diagnoses, and LOINC for lab tests.
Truven MarketScan^®^ Multi-state Medicaid (MDCD)	US Medicaid data from enrollees in multiple states	~17	1/1/2006-12/31/2013	MDCD contains adjudicated US health insurance claims for Medicaid enrollees from multiple states and includes hospital discharge diagnoses, outpatient diagnoses and procedures, outpatient pharmacy claims, and ethnicity and Medicare eligibility. Members maintain the same identifier even if they leave the system for a brief period. The dataset lacks lab data. Source data dictionaries include National Drug Codes (NDC) for prescriptions, CPT-4, HCPCs, ICD-9-CM for procedures, and ICD-9-CM for diagnoses.
Optum ClinFormatics^®^ (Optum)	US administrative health claims (United Healthcare)	~41	1/1/2006-12/31/2013	Optum is an adjudicated administrative health claims database for members with private health insurance, who are fully insured in commercial plans or in administrative services only (ASOs), Legacy Medicare Choice Lives (prior to January 2006), and Medicare Advantage (Medicare Advantage Prescription Drug coverage starting January 2006). The population is primarily representative of US commercial claims patients (0–65 years old) with some Medicare (65+ years old); ages are capped at 90 years. It includes data captured from administrative claims processed from inpatient and outpatient medical services and prescriptions as dispensed. The data also contain selected laboratory test results (those sent to a contracted thirds-party laboratory service provider) for a non-random sample of the population. Source data dictionaries include National Drug Codes (NDC) for prescriptions, CPT-4, HCPCs, ICD-9-CM for procedures, ICD-9-CM for diagnoses and LOINC for lab tests.
Clinical Practice Research Datalink (CPRD)	UK based general practice electronic health record	~11.6	1987-6/30/2013	CPRD is a governmental, not-for-profit research service, jointly funded by the NHS National Institute for Health Research (NIHR) and the MHRA, a part of the Department of Health, UK. CPRD consists of data collected from UK primary care for all ages. This includes clinical events, referrals, and lifestyle information gathered from primary care practices, along with medications as prescribed. Hospital, outpatient, and midwife data are included if primary care physician transcribes patient notes received from those facilities. Compliance varies by practice. Source data dictionaries include Gemscript for medications and Read for diagnoses, procedures, and additional types of clinical data.

All databases are licensed by Janssen Research and Development, LLC and were transformed to the OMOP Common Data Model v4 which has been described elsewhere [12, 13]. This study is based in part on data from the Clinical Practice Research Datalink obtained under licence from the UK Medicines and Healthcare products Regulatory Agency (MHRA). The protocol for this study (reference # 15_064) is provided with the submission (S1 ISAC) and was approved by the Independent Scientific Advisory Committee (ISAC). This study used only anonymized data in the Optum, CCAE, and MDCD databases without linkage to other databases or pursuit of further details through, e.g., chart reviews. The New England Institutional Review Board (IRB) has determined that use of databases in such studies does not involve human subjects and is therefore exempt from IRB approval.

#### Inclusion/exclusion criteria

Pregnancy episodes were included in the final cohort if the patient was female, between 12 and 55 years of age and had continuous enrollment during their pregnancy episode. Women were allowed to have multiple pregnancy episodes. Any outcome that did not have at least two associated pregnancy markers was excluded.

#### Code set for pregnancy markers and outcomes

The OMOP Common Data Model allows for standardization of clinical structure and content across all databases. All content (conditions, procedures and observations) in the OMOP Common Data Model are referred to as concepts. The OMOP Standard Vocabularies are used to understand and make use of these concepts [11, 14]. Native source codes are mapped to the dictionary that is considered standard for that domain (conditions, procedures, etc.) in the Standard Vocabularies. For instance, Read codes from CPRD and International Classification of Diseases, 9th Revision, Clinical Modification (ICD-9-CM) diagnosis codes from US claims databases are mapped to Systematized Nomenclature of Medicine—Clinical Terms (SNOMED-CT) concepts.

For this study, concept sets for pregnancy markers used to infer outcomes and pregnancy starts (S1 and S2 Tables) were developed using the OMOP Standard Vocabularies. Records referring to pregnancy outcomes and markers were identified from CPRD and Optum. CCAE and MDCR use the same source dictionaries as Optum. Pregnancy procedures were identified by ICD-9-CM, Current Procedural Terminology, 4th Edition (CPT-4), Healthcare Common Procedure Coding System (HCPCS), and SNOMED-CT concepts; conditions were identified by SNOMED-CT concepts; and observations were identified by Logical Observation Identifiers Names and Codes (LOINC) concepts. Observations in CPRD also included data from the ‘additional clinical details’ file.

Pregnancy concepts with 100 or more records in CPRD or Optum were categorized into the following outcome classification categories: live birth, stillbirth, abortion (spontaneous and induced), delivery, ectopic pregnancy, methotrexate exposure, surgery specific to ectopic pregnancy, and concepts highly associated with ectopic pregnancy per a disproportionality analysis. The disproportionality analysis was conducted to identify additional codes that were associated with ectopic pregnancy in CPRD, as it was common for only one ectopic pregnancy code to appear in general practice data and we required two for a valid episode in the algorithm. For the disproportionality analysis, prevalence of SNOMED-CT codes was assessed for females age 15 to 55 with a full year of observation in 2012 and prevalence of SNOMED-CT codes was also assessed during any pregnancy episode in CPRD up to 60 days after an ectopic pregnancy diagnosis. A curated list of concepts highly associated with ectopic pregnancy was derived from prevalence ratio higher than 5 in the ectopic pregnancy population vs. the 2012 female population. Pregnancy concepts in CPRD or Optum were used to infer pregnancy start for the following categories: premature birth, gestational age in weeks, last menstrual period, pregnancy confirmation, antenatal visit, pregnancy complication codes, threatened abortion, alpha fetoprotein screening tests, nuchal translucency ultrasound, fertility procedures that date conception, amenorrhea, contraceptive drug treatment, and urine pregnancy tests.

#### Outcome assessment and classification

The definition of a pregnancy episode for this work is the duration of time from the estimated last menstrual period date (pregnancy start) and the date of the pregnancy outcome (pregnancy end). The algorithm was devised to infer pregnancy start and end dates and outcome for each episode using observations in the data through a series of sequential steps. The algorithm in pseudocode form (based on SQL language used in algorithm) is shown in S1 and S2 Figs. Additional methods details are also provided in S1 Appendix.

In the first step of the algorithm, each pregnancy episode is classified into a pregnancy outcome. An outcome assessment hierarchy was applied, similar to that used in the studies by Mikolajcyzk et al [8] and Hornbrook et al [1], to classify episodes into outcome classes in sequential order. Hornbrook et al [1] found that live births and stillbirths in US claims data identified via ICD-9-CM diagnosis codes are most reliable so these are first in our hierarchy; procedure codes identifying abortions and deliveries were considered less reliable. Abortion procedure and diagnosis codes do not always provide guidance as to whether the abortion was spontaneous or induced so only one abortion category was created. Outcomes were assessed in this order:

- Live birth (at least one)
- Stillbirth
- Ectopic pregnancy
- Abortion
- Delivery record only

Independent outcome assessment per patient and classification steps were conducted as follows: starting with the first live birth record for a patient, the second live birth record was assessed to see if it occurred after the necessary time interval indicating a clinically plausible second pregnancy (S3 Table). S3 Table contains the minimum required duration between successive outcomes. These time windows depend on the specific outcomes under consideration and were adapted from the Hornbrook et al. [1] algorithm and clinically reviewed by two physicians (DF, AF), one of whom (AF) is an obstetrician. Consecutive live birth concepts occurring with more than the minimum required duration between them were classified as independent pregnancy outcomes.

Next all stillbirth records for each patient were assessed in order of occurrence. Each stillbirth record was compared to all live births and stillbirths already classified as outcomes for the patient, and was retained if it occurred outside the required window(s) of time between outcomes. If an antenatal visit or pregnancy confirmation record was found during the 42 day period after the stillbirth record date classified above then that stillbirth outcome did not become an independent pregnancy episode.

Next all ectopic pregnancy records for each patient were assessed in order of occurrence. Each ectopic pregnancy record was compared to all previously classified outcomes for the patient, and was retained if it occurred outside the required window(s) of time between outcomes. Ectopic pregnancy outcomes were also required to have during the subsequent 14 day period after the ectopic pregnancy record date: a methotrexate exposure, an ectopic pregnancy-related procedure, or a concept identified as highly associated with ectopic pregnancy from the disproportionality analysis. The ectopic pregnancy did not become an independent pregnancy episode if an antenatal visit or pregnancy confirmation record was found during the 42 day period after the ectopic pregnancy record date. Ectopic pregnancy outcome dates were reassigned to the last treatment date (methotrexate exposure or ectopic pregnancy-related procedure) in the 2 week period following the ectopic pregnancy record.

Next all abortion records for each patient were assessed in order of occurrence. Each abortion record was compared to all previously classified outcomes for the patient, and was retained if it occurred outside the required window(s) of time between outcomes. The abortion outcome did not become an independent pregnancy episode if an antenatal visit or pregnancy confirmation record was found during the 42 day period following the abortion record date. Abortion outcome dates were reassigned to the last abortion date in the 2 week period following the abortion record.

Lastly, delivery records for each patient were assessed in order of occurrence. Each delivery record was compared to all previously classified outcomes for a patient, and was retained if it occurred outside the required window(s) of time between outcomes. If an antenatal visit or pregnancy confirmation record was found during the 42 day period following the delivery date then that delivery outcome did not become an independent pregnancy episode. Delivery outcomes were re-classified as live births.

#### Start date estimation

In the second step, pregnancy episode start dates were estimated. For operating principles of the second step of the algorithm in pseudocode form see S2 Fig. In S4 Table the following outcome-specific term windows are defined:

- Maximum pregnancy term—amount of time to search back from the outcome for all pregnancy markers that can be used to estimate start date, i.e. 301 days for live births.
- Minimum pregnancy term—Shortest possible gestational period for each outcome, i.e. 161 days for live births.
- ‘Retry’ period: number of days initiation of a subsequent pregnancy episode is clinically possible after a pregnancy outcome.

All time windows were determined based on estimates in the literature specific to each outcome and through clinical review (DF, AF). Pregnancy start markers were considered for an episode if they created a pregnancy term length that was greater than the minimum term length and less than the maximum term length from S4 Table, and did not occur prior to the first occurring prior outcome date plus the retry period for that outcome.

A hierarchy of available pregnancy markers was chosen that reflects their potential accuracy to estimate start of the pregnancy episode. Pregnancy markers that directly provide gestational age such as last menstrual period (CPRD only), gestational age in weeks and fertility procedures that date conception were placed at the top of the hierarchy. Remaining markers considered for the hierarchy were screening tests administered during narrow gestational age windows such as nuchal ultrasounds, markers that indicate possible first antenatal visit for the pregnancy such as amenorrhea, and outcome-specific estimates. In order to select and place these remaining possible pregnancy start markers in the hierarchy, their start estimates were compared to start estimates from assisted conception procedures by pregnancy outcome in the Optum database. Among pregnancy episodes with assisted conception, the percentages of possible additional markers that were within two weeks prior or after the fertility procedure-based start date were calculated. This information was used to determine which markers were most proximal to the fertility procedure date in order to place them in the hierarchy for estimating starts in pregnancies without fertility procedures. The same analysis was done with nuchal ultrasounds substituted for assisted conception procedures. See S5 and S6 Tables for results.

We inferred the pregnancy episode start dates by identifying the first observed event amongst the pregnancy start marker hierarchy, as described below. If no pregnancy start markers were available to estimate pregnancy start, we used an outcome-specific estimate. After the highest ranking observed marker was identified the specified number of days was subtracted from the start marker date:

1. Last menstrual period date
2. Gestational age record date minus gestational age in days
3. Fertility procedure which dates conception record date minus 13 days
4. Nuchal translucency ultrasound record date minus 89 days
5. Alpha fetoprotein test record date minus 123 days
6. Amenorrhea record date minus 55 days
7. Urine pregnancy record date minus 55 days
8. Outcome-specific estimates (average gestational age estimate), adapted from Hornbrook et al. [1] and Margulis et al. [7]: The episode was classified as preterm if an associated preterm marker was found, otherwise it was classified as full-term. From S7 Table, the outcome-specific estimate was chosen based on outcome and term and subtracted from the outcome date to obtain a pregnancy start date. If the immediately prior outcome date plus retry period was greater than the estimated start, then pregnancy start became the prior outcome date plus the retry period.

We adjusted the final pregnancy start date estimated with amenorrhea, urine pregnancy and average gestational age estimates with contraceptive drug exposure and pregnancy confirmation markers if present; see S1 Appendix ‘Start Date Estimation’ section for additional details.

#### Illustrative patient example

**Fig 1:**
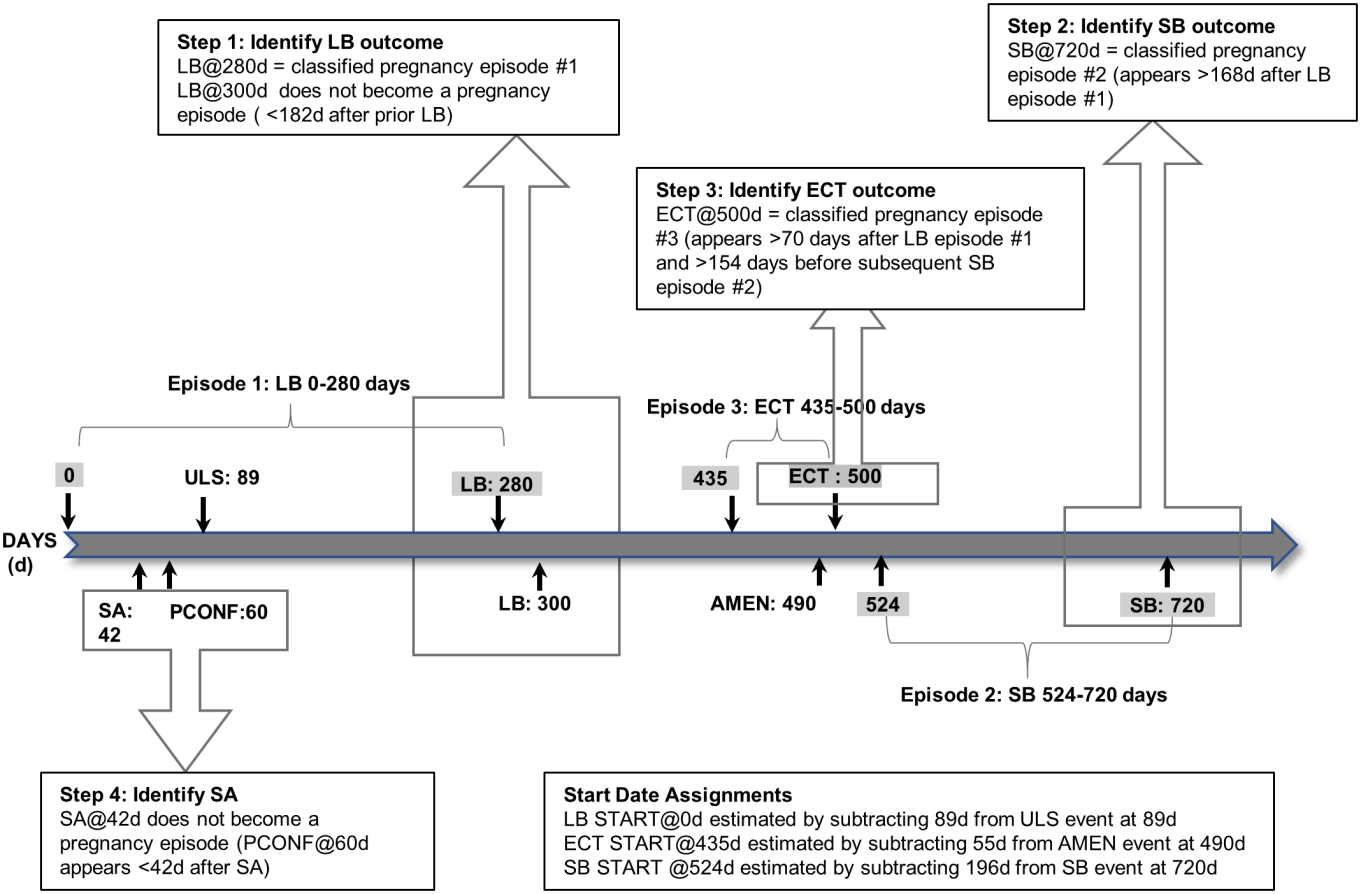
Illustration of steps performed by pregnancy episode algorithm for sample patient with three classified pregnancy episodes and estimated start dates. LB: Live birth; SB: Stillbirth; ECT: Ectopic pregnancy; SA: Abortion; PCONF: Pregnancy confirmation marker; ULS: Nuchal ultrasound; AMEN: Amenorrhea record.

Fig 1 contains an illustration of the steps performed by the pregnancy episode algorithm for a sample patient resulting in three classified pregnancy episodes and estimated start dates.

### Construction of pregnancy episodes

All pregnancy episodes for the above-mentioned data sources were constructed. For each data source for all pregnancy episodes, we generated outcome-type proportions, distribution of start date estimation method used per pregnancy outcome, and distribution of length of pregnancy episodes and stratified each by start calculation method for live births.

#### Validation

Pregnancy episode validation efforts included review of 700 pregnancy episode profiles comprised of 50 CPRD and 50 Optum randomly drawn episodes for all 6 pregnancy outcomes. An additional 50 profiles of live birth episodes for each database were also reviewed. The reviewers included a team of epidemiologists familiar with observational data (AM, PR, DF, DG, YW, JP, HP); three out of seven were not involved in creation of the pregnancy episode algorithm (YW, JP, HP). CPRD and Optum were validated with electronic patient profiles in order to assess the algorithm on both an electronic medical record database (CPRD) and an administrative claims database (Optum). Optum was chosen from the three administrative claims databases due to the size of the database and its generalizability. Like Optum, CCAE is a US employer-based administrative claims database with the same source dictionaries. MDCD uses the same source dictionaries as Optum as well, but contains a different population. To limit the resource burden of patient profile review and validation efforts, we chose all patient profiles for review from the two most disparate databases for patient profile validation, Optum and CPRD, a claims database and an EHR database, respectively. Optum only was chosen for assisted conception validation because assisted conception records are not consistently entered in the CPRD.

The data records that populated the pregnancy episode profiles originated from the CPRD or Optum databases and present a time-ordered snapshot of clinical care received around the time of the pregnancy episode. No medical chart review or abstraction was performed for this validation. All data records (conditions, procedures, observations and drug exposures) for each patient were included in electronic patient profiles ordered by days from the inferred pregnancy episode start, along with the algorithm-derived pregnancy episode start and outcome date, outcome chosen for the episode and possible start dates identified by the algorithm from the hierarchy above. To assess operating characteristics of the algorithm, reviewers were asked to answer six survey questions for each pregnancy episode which assessed different operating characteristics of the algorithm:

1. Was the patient pregnant during this episode?
2. Is the outcome classified correctly?
3. If the outcome is not classified correctly what should it be?
4. Is the outcome date assigned correctly?
5. If no, how many days after the algorithm inferred start should the outcome occur?
6. What is your estimated start of pregnancy (days from algorithm inferred start, 0 means you agree with inferred start)

For each data source, proportions of episodes which reviewers believed represented a pregnancy, had the correct outcome, and had the correct outcome date were generated by outcome. Reviewer-preferred start date within seven days before or after the algorithm calculated start, and reviewer preferred outcome date within seven days before or after the algorithm calculated outcome date were also generated by outcome. The distribution of reviewer preferred outcomes for incorrect algorithm outcomes, the distribution of reviewer estimated pregnancy start difference in days from the algorithm start (< -14, -8 thru -14, -1 thru -7, 0, 1 through 7, 8 through 14 and > 14 days) and the distribution of the reviewer preferred outcome date difference in days from the algorithm outcome date (< -14, -8 through -14, -1 through -7, 0, 1 through 7, 8 through 14 and > 14 days) were determined, all stratified by outcome, data source and method used for pregnancy start calculation as applicable.

A sensitivity analysis was performed to validate the algorithm start estimation against assisted conception fertility procedures that in theory accurately date conception for all outcomes. Within the population of Optum pregnancy episodes with these procedures, we compared pregnancy starts calculated using fertility procedures to start estimation using the next highest marker in our hierarchy. Optum was chosen for this analysis because assisted conception procedures are not entered consistently in the CPRD, and for the same reasons stated above the Optum results were intended to be generalized to CCAE and MDCD.

## Results

### Characterization of pregnancy episodes

A total of 885,608 pregnancy episodes were found in CPRD, 1,087,626 in Optum, 3,278,013 in CCAE and 518,112 in MDCD. Distributions of pregnancy outcome types were similar across all data sources: live birth proportions were 71.59%, 72.84%, 72.85% and 79.83%, abortion 27.16%, 25.59%, 25.49% and 18.05%, stillbirth 0.45%, 0.47%, 0.45% and 0.78% and ectopic pregnancies 0.79%, 1.10%, 1.21% and 1.35% in CPRD, Optum, CCAE and MDCD, respectively.

**Fig 2:**
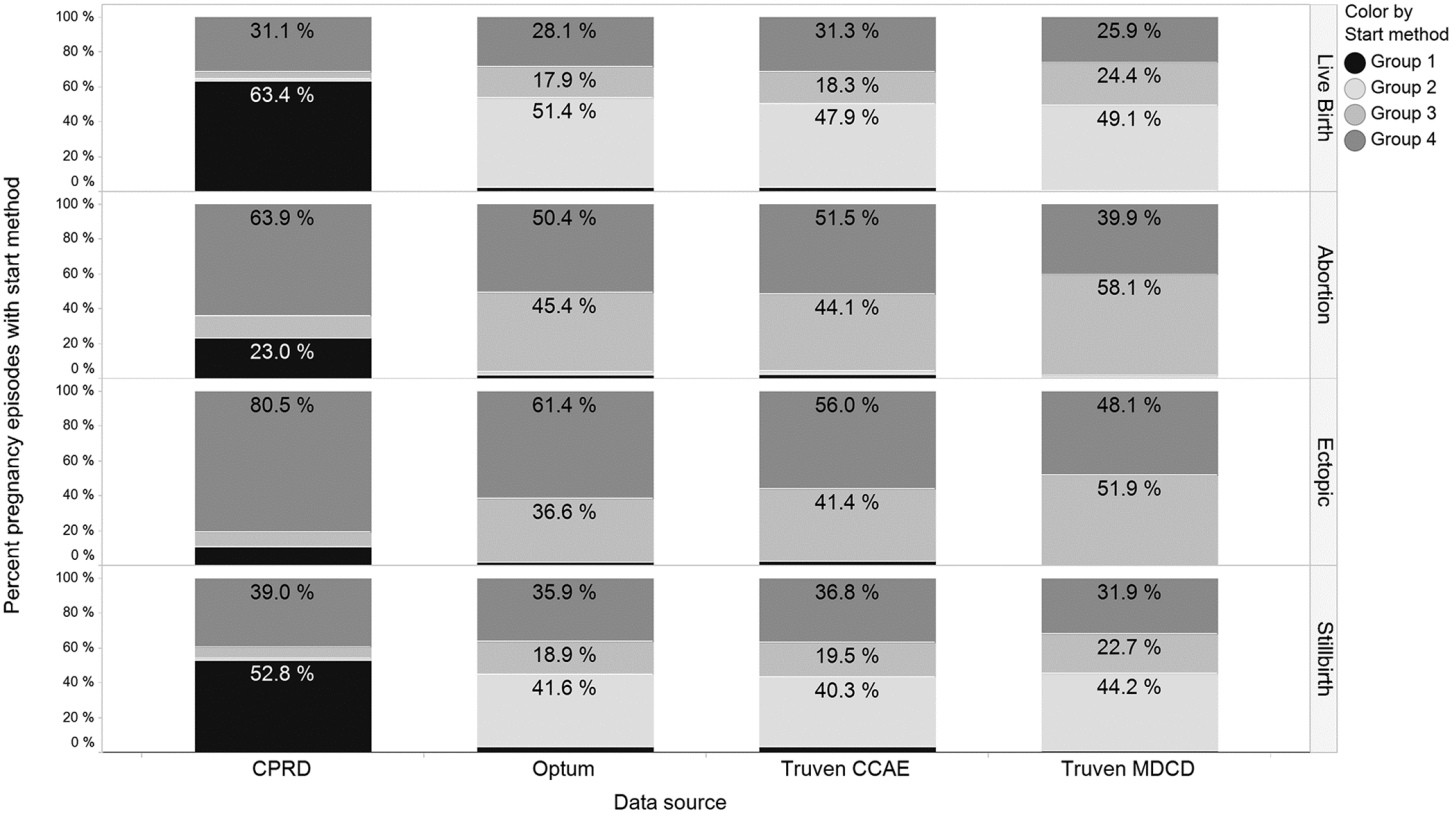
Distribution of pregnancy algorithm start estimation method groups for all pregnancy episodes from each data source (CPRD, Optum, Truven CCAE, and Truven MDCD) by outcome (abortion, ectopic pregnancy, live birth, and stillbirth). Legend for start estimation methods in stacked bars: Group 1 = last menstrual period, gestational age record, fertility procedure; Group 2 = alpha-fetoprotein test, nuchal ultrasound; Group 3 = amenorrhea record, urine pregnancy test record; Group 4 = average gestational age estimate.

In Fig 2 for CPRD, 63.4% of live birth episodes used a last menstrual period, gestational age record or fertility procedure record for start estimation; while 31.1% estimated start using the average gestational age estimate. A total of 51.4% of live births in Optum used the date of an alpha fetoprotein test or nuchal ultrasound to estimate start date; 17.9% used an amenorrhea or urine pregnancy test record; 28.1% used the average gestational age estimate.

Optum and Truven median pregnancy episode length of 277 was attained for live births using nuchal ultrasound dates for start estimation; CPRD had median length of 282. Truven median episode length of 111 was attained for abortions using nuchal ultrasound dates for start estimation; Optum 113; CPRD 102. Truven median episode length of 58 was attained for ectopic pregnancies using nuchal ultrasound dates for start estimation; Optum 63. Truven median episode length of 187 was attained for stillbirths using nuchal ultrasound dates for start estimation; Optum 185; CPRD 207 (S8 Table).

**Fig 3:**
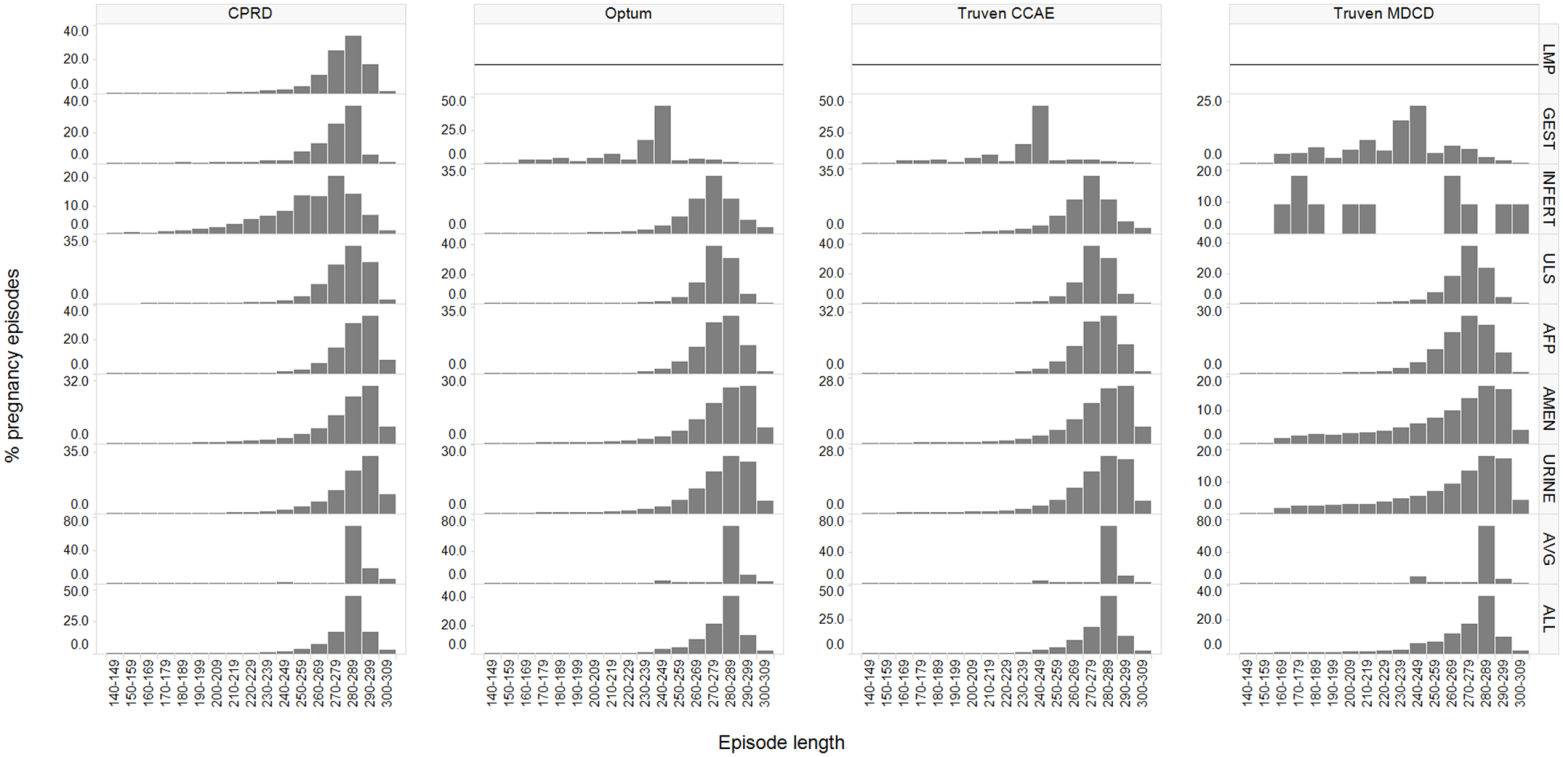
Distribution of duration of all pregnancy episodes, with episode lengths shown in 10 day increments for live births, stratified by method of pregnancy start estimation and data source (CPRD, Optum, Truven CCAE, and Truven MDCD). Legend for start estimation methods referenced in row panels: LMP = last menstrual period GEST = gestational age record INFERT = fertility procedure ULS = nuchal ultrasound AFP = alpha-fetoprotein test AMEN = amenorrhea record URINE = urine pregnancy record AVG = gestational age estimate ALL = all start estimation methods combined.

In Fig 3 for Optum, pregnancy episode lengths calculated with alpha fetoprotein test markers were distributed as follows: 270–279 days (27.1%); and 280–289 days (30.4%). In CPRD, pregnancy episode lengths calculated with last menstrual period markers were distributed as follows: 270–279 days (25.3%); and 280–289 days (33.7%). Amenorrhea and urine pregnancy markers created more episodes longer than > = 290 days; for instance, in Optum, 24.7% of episodes were 290–299 days long using amenorrhea records to estimate start of pregnancy, while 6.7% of episodes were 290–299 days long when nuchal ultrasound dates were used.

### Validation results

**Fig 4:**
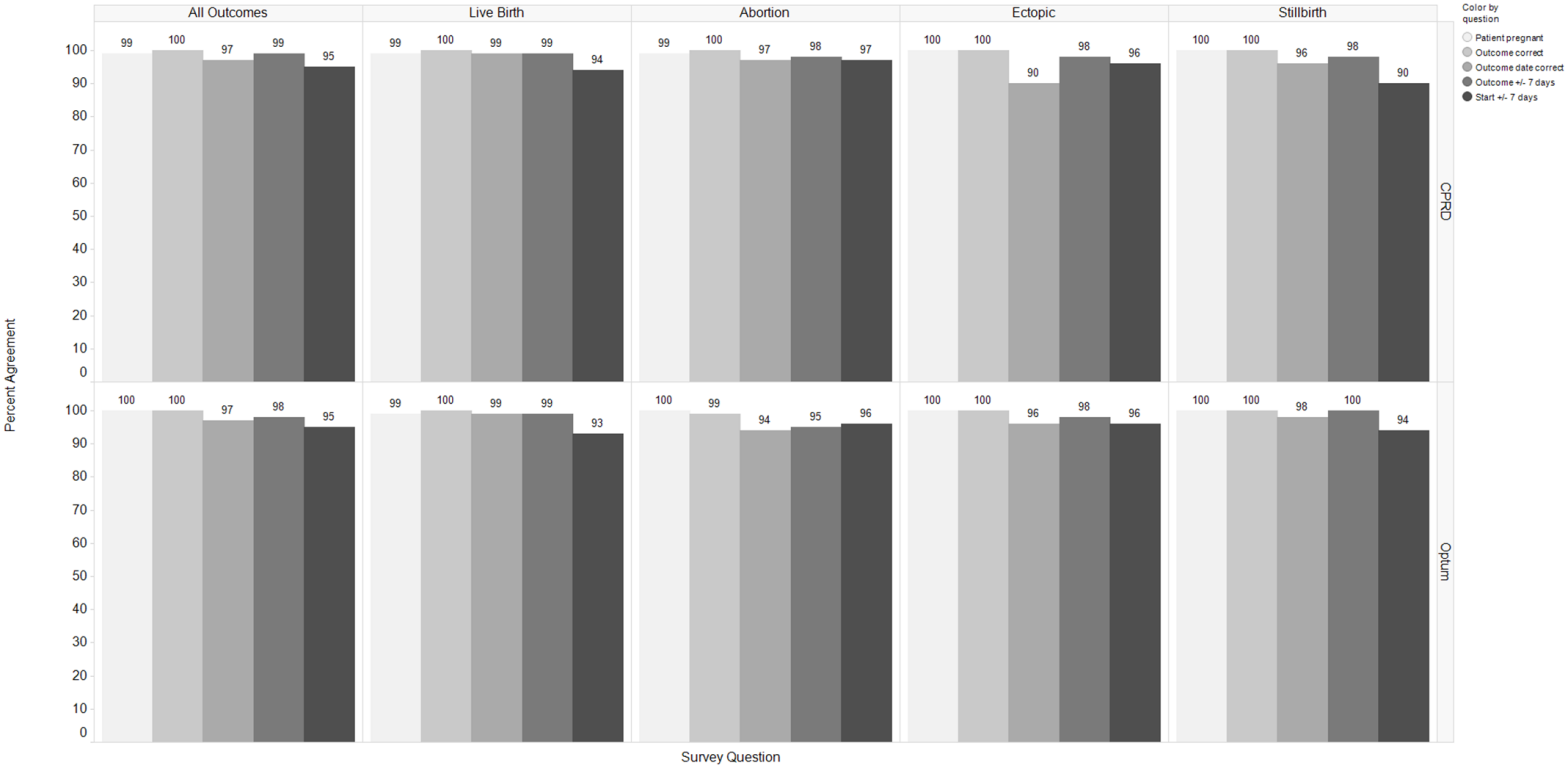
Performance rating for five operating characteristics of algorithm (survey questions 1,2,4–6) from electronic profile review survey.

Performance was rated for six different operating characteristics of the algorithm from survey question responses generated by electronic profile review of the pregnancy episodes. The survey questions 1) Was the patient pregnant during this episode? and 2) Is the outcome classified correctly? were answered affirmatively for 99–100% of all outcome and database categories. Survey question 3) If the outcome is not classified correctly what should it be? was only answered affirmatively for one Optum episode. The algorithm classified this episode as an abortion while the reviewer classified the episode as an ectopic pregnancy outcome. Remaining survey question results can be found in Fig 4.

**Fig 5:**
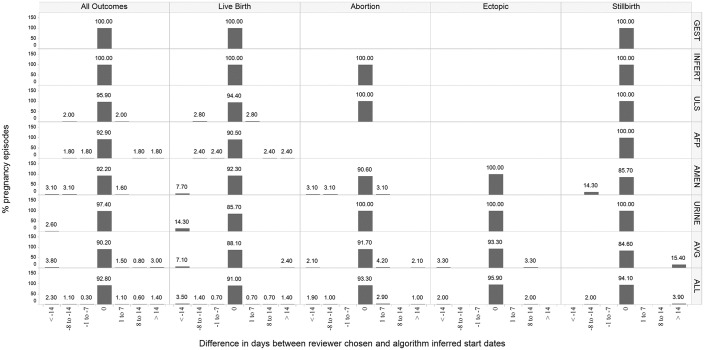
The distribution of reviewer estimated pregnancy start difference in days from the algorithm start (< -14, -8 thru -14, -1 thru -7, 0, 1 through 7, 8 through 14 and > 14 days) in the Optum database, stratified by pregnancy outcome (all outcomes, live birth, abortion, ectopic pregnancy, and stillbirth) and method used for pregnancy start estimation. Legend for start estimation methods referenced in row panels: LMP = last menstrual period GEST = gestational age record INFERT = fertility procedure ULS = nuchal ultrasound AFP = alpha-fetoprotein test AMEN = amenorrhea record URINE = urine pregnancy record AVG = average gestational age estimate ALL = all start estimation methods combined *If percentages do not add up to 100%, reviewer indicated difference in days from the algorithm could not be determined.

**Fig 6:**
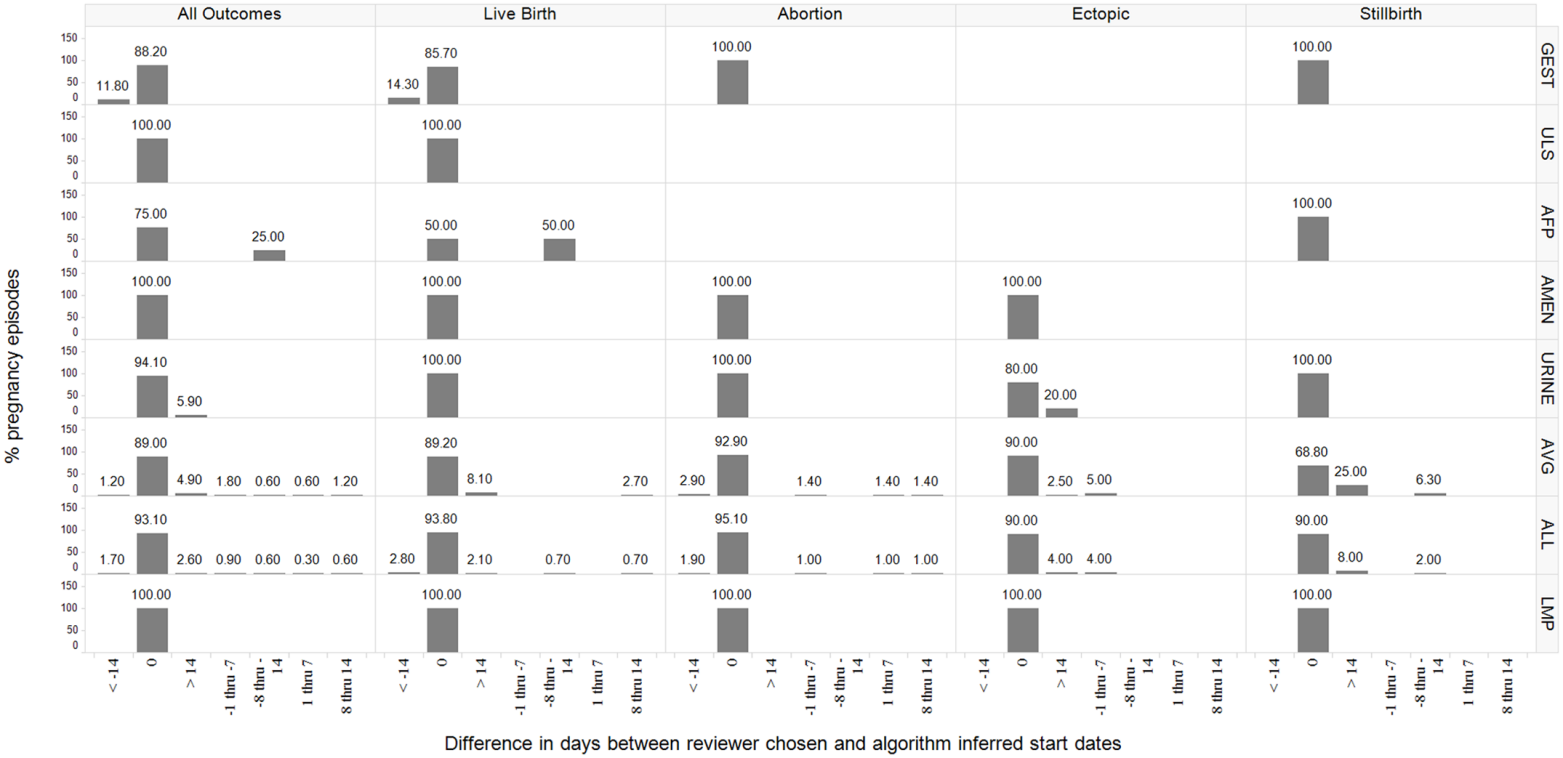
The distribution of reviewer estimated pregnancy start difference in days from the algorithm start (< -14, -8 thru -14, -1 thru -7, 0, 1 through 7, 8 through 14 and > 14 days) in the CPRD database, stratified by pregnancy outcome (all outcomes, live birth, abortion, ectopic pregnancy, and stillbirth) and method used for pregnancy start estimation. Legend for start estimation methods referenced in row panels: LMP = last menstrual period GEST = gestational age record INFERT = fertility procedure ULS = nuchal ultrasound AFP = alpha-fetoprotein test AMEN = amenorrhea record URINE = urine pregnancy record AVG = average gestational age estimate ALL = all start estimation methods combined *If percentages do not add up to 100%, reviewer indicated difference in days from the algorithm could not be determined.

For all outcomes, the proportion of episodes in Optum with 0 days difference between reviewer estimated starts and algorithm starts were: using alpha fetoprotein screening test (92.9%), amenorrhea diagnosis (92.2%), average gestational age estimates (90.2%), nuchal ultrasound (95.9%) and urine pregnancy test (97.4%) (Fig 5). In CPRD, the last menstrual period pregnancy marker performed best when compared to reviewer estimated pregnancy starts, with 100% algorithm-reviewer agreement with 0 days difference. The average gestational age estimate method yielded 89% pregnancy episodes with 0 days difference and gestational age records in weeks yielded 88.2% with 0 days difference (Fig 6).

Table 2 indicates that for all CPRD outcomes, the algorithm chose the outcome date on the same day as the reviewer preferred outcome date 96.8% of the time. In Optum for all outcomes, the algorithm chose the outcome date on the same day as the reviewer preferred outcome date 97.1% of the time. Table 3 shows results from the Optum validation sensitivity analysis, which revealed that for the population of pregnancy episodes that had associated assisted conception fertility procedures (19,656 episodes), 48.9% of live births starts that were estimated with the next highest marker in the hierarchy were within seven days before or after the pregnancy start that was estimated with the fertility procedure.

**Table 2:** End of pregnancy episode date accuracy: Reviewer chosen date categorized by difference from algorithm derived date.

Data Source	Outcome	< -14 days N(%)	-8 thru -14 days N(%)	-1 thru -7 days N(%)	0 days N(%)	1 thru 7 days N(%)	8 thru 14 days N(%)	> 14 days N(%)
CPRD	All Outcomes	4 (0.6)	6 (0.9)	2 (0.3)	674 (96.8)	10 (1.4)	0	0
	Live Birth	2 (0.7)	0	0	286 (98.6)	2 (0.7)	0	0
	Ectopic	0	2 (2)	2 (2)	90 (90)	6 (6)	0	0
	Abortion	2 (1)	2 (1)	0	202 (98.1)	0	0	0
	Stillbirth	0	2 (2)	0	96 (96)	2 (2)	0	0
Optum	All Outcomes	4 (0.6)	6 (0.9)	4 (0.6)	678 (97.1)	0	2 (0.3)	4 (0.6)
	Live Birth	0	0	0	286 (99.3)	0	0	2 (0.7)
	Ectopic	2 (2)	0	2 (2)	94 (95.9)	0	0	0
	Abortion	2 (1)	6 (2.9)	0	198 (94.3)	0	2 (1)	2 (1)
	Stillbirth	0	0	2 (2)	100 (98)	0	0	0

**Table 3:** Optum validation sensitivity analysis: Algorithm chosen pregnancy start categorized by difference in either direction from infertility procedure derived start.

Pregnancy outcome	Days difference in either direction	Pregnancy episodes	% agreement
Abortion	< = 7 days	1090	20.03
Abortion	8–14 days	890	16.35
Abortion	> 14 days	3462	63.62
Ectopic Pregnancy	< = 7 days	96	39.51
Ectopic Pregnancy	8–14 days	90	37.04
Ectopic Pregnancy	> 14 days	57	23.46
Live Births	< = 7 days	6749	48.91
Live Births	8–14 days	3366	24.39
Live Births	> 14 days	3684	26.7
Stillbirth	< = 7 days	54	31.4
Stillbirth	8–14 days	26	15.12
Stillbirth	> 14 days	92	53.49

## Discussion

In this study, we developed an algorithm to infer pregnancy episodes and their outcomes from observational data, and applied the algorithm across the CPRD, Optum, Truven CCAE and MDCD databases. Distributions of pregnancy outcome types were similar across all four data sources and pregnancy episode lengths found were as expected for all outcomes. The validation was performed via review of electronic patient profiles and completion of a survey by personnel familiar with observational data. Highest agreement between reviewer chosen and algorithm operating characteristics was achieved for questions assessing pregnancy status and accuracy of outcome category with 99–100% agreement for all data sources. Outcome date agreement within seven days in either direction ranged from 95–100%, while start date agreement within seven days in either direction ranged from 90–97%. In Optum, a total of 73% of algorithm estimated starts for live births and a total of 77% of ectopic pregnancies were in agreement with fertility procedure estimated starts in our sensitivity analysis within two weeks in either direction. However, stillbirth estimated starts agreed 47% of the time, and abortion 36%. Therefore, according to the patient profile validation exercise, start estimations for all outcomes were fairly accurate while the sensitivity analysis found abortion and stillbirth start estimations less accurate than live birth and ectopic pregnancy. We believe this is acceptable accuracy for live births and ectopic pregnancies considering the potential sample size and prescription accuracy observational data offers and the challenge that repeat delivery procedure billings and rule-out diagnoses create in the electronic record, however there may be less accuracy around stillbirth and abortion start date estimations.

### Strengths and limitations

With this study we attempted to build on prior pregnancy algorithm studies using observational data in the literature by incorporating relevant features across the spectrum into a single algorithm. To the best of our knowledge, there is no prior algorithm that incorporates all of the following features: assessment of multiple pregnancy outcomes (live birth, stillbirth, abortion and ectopic pregnancy), start estimation leveraging a hierarchy of available start markers, internal validation using electronic patient profiles with consideration of the patient’s entire clinical experience, and validation of the generic algorithm across multiple, disparate data sources.

Various algorithm limitations and caveats are important to note. For instance, in order to classify pregnancy outcomes in the data, our algorithm referred to a table containing the shortest period of time that can occur between any two outcomes. If an outcome being assessed was too close to another outcome that was already inferred by the algorithm (either the outcome was higher in the outcome hierarchy or was the same outcome with an earlier date) then that outcome was discarded. In this way, multiple billings related to the same health care episode were not counted as separate pregnancy episodes. This was necessary because multiple clinical care records that are billed separately on different dates for various aspects of delivery care are common in observational data. We found in our analysis that administrative billing databases such as Optum and CCAE are prone to this scenario; electronic health records such as CPRD generally record fewer diagnoses and procedures around a single health care episode. It is possible valid outcomes with record dates too close in days per S3 Table would not be captured.

A second challenging clinical care situation frequently recorded in the data included a missed abortion rule-out diagnosis prior to a preterm live birth. Rule-out diagnoses are billings for tests ordered to diagnosis symptoms, where the suspicion rather than the confirmation of the diagnosis is recorded to justify reimbursement. Again, this scenario is more common in Optum and CCAE; in CPRD codes for ‘working diagnoses’ can be chosen instead which were not utilized by our algorithm. Excluding candidate abortion or ectopic pregnancy episodes with pregnancy confirmation within 42 days after also helped to eliminate these rule-out diagnoses. However, it is possible some rule-out diagnoses were considered by the algorithm to be valid outcomes.

Initially, an attempt was made to create two abortion outcomes: spontaneous and induced. After validation with patient profiles, we determined the algorithm could not classify an abortion as induced or spontaneous with acceptable accuracy; a proportion of abortion diagnosis and procedure concepts were sufficiently ambiguous that a determination was difficult to make during initial creation of outcome code set and in certain cases mappings from native source dictionaries to Common Data Model concepts created additional ambiguity.

Also, ectopic pregnancies were classified only if treatment or a record type identified by the disproportionality analysis was found within two weeks after initial diagnosis. Scholes et al. found that ≥2 visits with an ectopic pregnancy code (procedures and diagnoses) within 180 days is highly predictive of a true ectopic pregnancy based on chart review [15]. Our time window of two weeks was more restrictive since treatment is commonly administered in an emergency fashion for ectopic pregnancy. However, it is possible some true ectopic pregnancy episodes were missed by our algorithm because of delayed treatment.

The requirement that an episode have two pregnancy records, which was meant to exclude historical references to pregnancy outcomes, may also have excluded legitimate episodes with only a single outcome record and no other pregnancy markers. In CPRD, more pregnancy episodes were discarded for this reason. It is likely this occurred because primary care physicians were not transcribing pregnancy care into the primary record and only recording birth outcomes. In Optum and CCAE, multiple billings for the same health care episode made exclusion of the pregnancy episode due to less than one pregnancy care record less common. Finally, our algorithm was not designed to distinguish between singleton and multiple births. The algorithm outcome hierarchy utilized will classify a delivery with at least one live birth as a live birth outcome; if a live birth occurred along with a stillbirth in a multiple birth, the outcome would be classified as a live birth.

From Fig 2 it can be seen that pregnancy care records used to estimate start of pregnancy in CPRD have a different distribution than those used to estimate start in Optum, CCAE and MDCD. In CPRD, 52.8% of pregnancy starts were estimated using last menstrual period (LMP), gestational age record, fertility procedure, all of which were rated highest in the hierarchy of available pregnancy markers. It is likely CPRD pregnancy start estimation was more accurate because of the high prevalence of LMP records available in the data and lack of LMP data in Optum, CCAE and MDCD, though the patient profile survey does not reflect this. Maximum pregnancy terms (i.e. 301 days for live births) were used to search backward from the outcome date for codes that could help define pregnancy starts, so earlier pregnancy-related codes would not be found by the algorithm. Margulis et al. also found that dates based on screening tests were less accurate than average gestational age estimates to estimate pregnancy start when compared with delivery discharge records for live births [7]. The analysis we performed to determine the hierarchy of available pregnancy markers may have had different results because we defined minimum and maximum pregnancy terms allowed in the algorithm so that screening tests performed well outside the clinical guideline windows were not used to estimate pregnancy start.

A trade-off was made with the elevation of amenorrhea and urine pregnancy test markers above average gestational age estimates in our hierarchy, so that adverse outcomes such as abortion and stillbirth had potentially increased start date accuracy and live births and ectopic pregnancy had potentially decreased accuracy when amenorrhea and urine pregnancy markers were used. This would have less of an effect in CPRD which had few of these markers associated with live births and more of a possible effect in Optum with 17.9% of live birth episodes using amenorrhea and urine pregnancy markers to estimate start. In Optum, Fig 3 illustrates that episodes with amenorrhea and urine pregnancy markers used as start estimation had a larger proportion of post-term pregnancies than would be expected. It is possible a percentage of women sought care a week or so earlier than the estimated eight weeks after last menstrual period.

Given that our algorithm uses a hierarchical approach, combining many of the criteria that have been previously used to infer pregnancy episodes, it is expected that our approach yields more robust capture of estimated pregnancy episodes than has been previously documented. Internal validation of the algorithm by multiple clinicians and epidemiologists using a sample of patient profiles showed high validity and reliability across both EHR and administrative claims databases. External patient charts may have been available for some of the pregnancy episodes, but the chart costs and personnel hours required exceeded study budget allocation. While both internal and external validation methods are widely used in observational studies, there are advantages and limitations to both with no clear consensus on a gold standard [16,17]. Our novel approach enabled our algorithm to be compared to expert opinion of clinicians’ and epidemiologists’ determination of a true pregnancy episode after viewing all data elements within each patient record. This was determined by the authors to be the most appropriate validation measure as it is a direct measure of how well a derived algorithm can accurately sort through data elements to identify those that indicate pregnancy. A better understanding of whether or not observational databases themselves contain accurate information on pregnancy occurrences could potentially be achieved by using external validation, however, this is outside the scope of our paper.

We acknowledge several limitations inherent to our internal validation method. For instance, the internal review done on pregnancy episodes with algorithm estimates visible to reviewers may have caused reviewers to agree with the algorithm if there was a lack of claims or EHR records to provide guidance, even if a shorter or longer pregnancy term may have been a more reasonable estimation for that episode. This may have increased reviewer agreement and caused the electronic review to overestimate accuracy, especially for outcomes like AB, ECT and SB which often did not have early pregnancy start markers. Though it was not possible for our study, comparison with an external data source, such as hospital birth records, would have mitigated this issue for represented outcomes. Also, four out of seven reviewers were involved in creation of the pregnancy algorithm and may have anchored their validation on the same information the algorithm utilized. Three reviewers were unaware of the methodology used in the algorithm.

Though a total of 73% of Optum algorithm estimated starts for live births and 77% of algorithm estimated starts for ectopic pregnancies were in agreement with fertility procedure estimated starts in our sensitivity analysis within two weeks in either direction, there was less agreement for abortion and stillbirth. This was possibly due to misclassification of infertility treatment date, but it is more likely that in the absence of pregnancy start markers, gestational age estimates are more accurate for full-term live births (280 days) and ectopic pregnancies (56 days) than for stillbirths (196 days) and abortions (70 days); the possible ranges of episode length for abortions and stillbirths are wider than for live births and stillbirths making estimation more difficult. This is potentially aggravated by the fact that stillbirths and spontaneous abortions may not be clinically recognized for a period of time after the event.

When considering the larger research agenda of establishing exposure effects in pregnancy, there are advantages and limitations inherent to observational data. The pregnancy algorithm does allow us to examine length of pregnancy as an outcome to evaluate preterm birth, however, other outcomes associated with the child, such as malformations, are data captured in the child’s record, and require creation of a mother-child linkage which is not in scope for defining start/end of pregnancy. Also, exposure may be under- or overestimated for a number of reasons. Administrative claims databases reflect billed insurance claims. Recorded diagnoses and procedures are the basis of reimbursement and the checks and requirements applied to such information may serve to improve its accuracy. The exposure information that is captured is based on prescriptions (EHRs such as CPRD) or dispensings (claims data) rather than on recall, though there is no way to be certain the patient actually took the medication that was prescribed or dispensed. Conditions must also be diagnosed, and in the case of administrative claims databases, billed for. If a patient has a visit for more than one condition, each condition may not be recorded or billed separately, which may lead to under-diagnosis in the database. Services that providers know in advance will be denied will be inconsistently submitted as bills, and therefore, inconsistently recorded. Some additional information is limited, such as information on severity, physiology, and reason for testing. Covered services for which claims are not submitted are not included, such as immunizations provided through grocery-store immunization clinics. Rule-out diagnoses are another inherent limitation to claims databases, and have been discussed previously and addressed within our algorithm. Primary care databases such as CPRD have inconsistent recording of clinical care in hospital and specialist or outpatient settings. Despite these limitations, the advantages of observational data outlined earlier allow for unique opportunities to longitudinally evaluate the association between exposures and outcomes, particularly in hard to reach and understudied populations such as pregnant women.

## Conclusion

In this study, we developed an algorithm to infer pregnancy episodes and their outcomes and applied the algorithm across four observational databases. To the best of our knowledge, there is no prior algorithm that incorporates all of the following features: assessment of multiple pregnancy outcomes (live birth, stillbirth, abortion and ectopic pregnancy), start estimation leveraging a hierarchy of available start markers, internal validation using electronic patient profiles with consideration of the patient’s entire clinical experience, and validation of the generic algorithm across multiple, disparate data sources.

Validation survey results were highly positive, with agreement at least 90% of the time with reviewer chosen characteristics. During algorithm design, care was taken to avoid defining rule-out diagnoses and repeat billings as separate pregnancy episodes and validation survey results indicated these situations were handled correctly for our sample. Our examination of all pregnancy markers that could be used to estimate start of pregnancy and establishment of a hierarchy was successful as evidenced by pregnancy terms that generally align with expectations, excepting term lengths in episodes that used amenorrhea and urine pregnancy tests as start estimation. Additionally, agreement of reviewer chosen pregnancy starts with algorithm chosen pregnancy starts in the electronic profile validation was high. The validation sensitivity analysis using fertility procedures that date conception also suggests an acceptable level of accuracy is reached in start date estimation by the algorithm for live births and ectopic pregnancies. An algorithm to infer live birth and ectopic pregnancy episodes and outcomes can be applied to multiple observational databases with acceptable accuracy for further epidemiologic research. Less accuracy was found for start date estimations in stillbirth and abortion outcomes in our sensitivity analysis, which may be expected given the nature of the outcomes.

## Supporting information

S1 Table OMOP standard vocabulary categorized pregnancy concept sets. (XLSX)

S2 Table Database source dictionary categorized pregnancy codes. (XLSX)

S3 Table Minimum number of days required to identify separate pregnancy outcomes. (XLS)

S4 Table Time windows in days necessary to calculate pregnancy episode start date. (XLS)

S5 Table Percentage of pregnancy episodes with algorithm start date estimation within 2 weeks prior or after infertility procedure estimation for various pregnancy markers. (XLS)

S6 Table Percentage of pregnancy episodes with algorithm start date estimation within 2 weeks prior or after nuchal ultrasound for various pregnancy markers. (XLS)

S7 Table Outcome specific gestational age estimates in days. (XLS)

S8 Table Descriptive statistics duration of all pregnancy episodes, stratified by pregnancy outcome, method of pregnancy start estimation and data source (CPRD, Optum, Truven CCAE, and Truven MDCD). (XLS)

S1 Appendix Supplementary pregnancy episode algorithm method details. (DOCX)

S1 Fig Pregnancy episode algorithm pseudocode: Outcomes assessment. (DOCX)

S2 Fig Pregnancy episode algorithm pseudocode: Episode start estimation. (DOCX)

S3 Fig Alternative illustrative patient example. (DOCX)

S1 Data Fig 2 distribution of pregnancy algorithm start estimation method groups for all pregnancy episodes from each data source (CPRD, Optum, Truven CCAE, and Truven Medicaid) by outcome (abortion, ectopic pregnancy, live birth, and stillbirth). (TXT)

S2 Data Fig 3 distribution of duration of all pregnancy episodes, with episode lengths shown in 10 day increments for live births, stratified by method of pregnancy start estimation and data source (CPRD and Optum). (TXT)

S3 Data Fig 4 performance rating for five operating characteristics of algorithm (survey questions 1,2,4–6) from electronic profile review survey. (TXT)

S4 Data Fig 5 the distribution of reviewer estimated pregnancy start difference in days from the algorithm start (< -14, -8 thru -14, -1 thru -7, 0, 1 through 7, 8 through 14 and > 14 days) in the Optum database, stratified by pregnancy outcome (all outcomes, live birth, abortion, ectopic pregnancy, and stillbirth) and method used for pregnancy start estimation. (TXT)

S5 Data Fig 6 the distribution of reviewer estimated pregnancy start difference in days from the algorithm start (< -14, -8 thru -14, -1 thru -7, 0, 1 through 7, 8 through 14 and > 14 days) in the CPRD database, stratified by pregnancy outcome (all outcomes, live birth, abortion, ectopic pregnancy, and stillbirth) and method used for pregnancy start estimation. (TXT)

S1 ISAC Approved ISAC protocol reference # 15_064. (DOCX)
