# Supplementary material for: Inferring pregnancy episodes and outcomes within a network of observational databases
Source: PLoS One. 2018 Feb 1;13(2):e0192033. doi: 10.1371/journal.pone.0192033 (PMC5794136; doi:10.1371/journal.pone.0192033)
Supplement: S1 Appendix — (DOCX) [file pone.0192033.s009.docx]

## Pregnancy Episode Algorithm Additional Details

### Outcome classification

The definition of a pregnancy episode for this work is the duration of time from the estimated last menstrual period date (pregnancy start) and the date of the pregnancy outcome (pregnancy end). The algorithm was devised to infer pregnancy start and end dates and outcome for each episode using observations in the data through a series of sequential steps.

In the first step of the algorithm, all pregnancy episodes are classified into a pregnancy outcome. We applied an outcome assessment hierarchy to classify episodes into outcome classes in sequential order. Outcomes were assessed in this order:

- Live birth (at least one)
- Stillbirth
- Ectopic pregnancy
- Induced and spontaneous abortion
- Delivery record only

Starting with the first live birth record for a patient, the second live birth record was assessed to see if it occurred after the necessary time interval indicating a clinically plausible second pregnancy (see S3 Table). S3 Table contains the minimum required duration between successive outcomes (for example, a minimum of 182 days was required between two live birth outcomes for a person). These time windows can change based upon the specific outcomes under consideration and were clinically reviewed. Consecutive live birth concepts occurring with more than the minimum required duration were classified as independent pregnancy outcomes. Live birth concepts which occurred before the minimum required duration following a prior live birth concept were excluded from consideration. Once all live birth concepts were processed, each person would be classified as having one or more live birth episodes.

Next, all stillbirth records for a person were assessed in order of occurrence. Each stillbirth record was compared to live births and stillbirths already classified as outcomes for a patient, and were retained if they occurred outside the required window(s) of time between outcomes from S3 Table. For example, if a patient had a stillbirth record more than 168 days after a live birth record classified in the first step and a second live birth record classified in the first step occurred more than 182 days after the stillbirth then the stillbirth record would be retained as an outcome for the patient because the minimum required amount of days between outcome types had elapsed between the prior and subsequent outcomes. Stillbirth outcomes classified in the current step were also considered if they occurred prior to the stillbirth record in consideration. For example, a patient with a stillbirth record and another stillbirth record previously classified in the current step more than 168 days prior and a live birth outcome classified in the previous step more than 182 days after would have two classified stillbirth outcomes and one live birth outcome. If an antenatal visit or pregnancy confirmation record was found within 42 days of a stillbirth outcome classified above then that stillbirth outcome was not retained.

Next, all ectopic pregnancy records for a person were assessed in order of occurrence. Each ectopic pregnancy record was compared to live births, stillbirths, and ectopic pregnancies already classified as outcomes for a patient, and were retained if they occurred outside the required window(s) of time between outcomes from S3 Table. For example, if a patient had an ectopic pregnancy record more than 70 days after a live birth record classified in the first step and a stillbirth record classified in the second step occurred more than 154 days after the ectopic pregnancy then the ectopic pregnancy record would be retained as an outcome for the patient. Ectopic pregnancy outcomes classified in the current step were also considered if they occurred prior to the ectopic pregnancy record in consideration. For example, a patient with an ectopic pregnancy record and another ectopic pregnancy record previously classified in the current step more than 56 days prior and a live birth outcome classified in the first step more than 168 days after would have two classified ectopic pregnancy outcomes and one live birth outcome. Ectopic pregnancy outcomes were also required to have within 14 days of the ectopic pregnancy record a methotrexate exposure, an ectopic pregnancy-related procedure, or a concept identified as highly associated with ectopic pregnancy via a disproportionality analysis. If an antenatal visit or pregnancy confirmation record was found within 42 days of an ectopic pregnancy outcome classified above then that ectopic pregnancy outcome was not retained. Ectopic pregnancy outcome dates were reassigned to the last treatment date (methotrexate exposure or ectopic pregnancy-related procedure) within 2 weeks of the ectopic pregnancy record.

Next, all spontaneous and induced abortion records for a person were assessed in order of occurrence. Each abortion record was compared to live births, stillbirths, ectopic pregnancies, and abortions already classified as outcomes for a patient, and were retained if they occurred outside the required window(s) of time between outcomes from S3 Table. For example, if a patient had an abortion record more than 70 days after a live birth record classified in the first step and a stillbirth record classified in the second step occurred more than 154 days after the abortion then the abortion record would be retained as an outcome for the patient. Abortion outcomes classified in the current step were also considered if they occurred prior to the abortion record in consideration. If an antenatal visit or pregnancy confirmation record was found within 42 days of an abortion outcome classified above then that abortion outcome was not retained. Abortion outcome dates were reassigned to the last spontaneous or induced abortion date within 2 weeks of the abortion record. Maximum and minimum allowed terms were different for spontaneous and induced abortions see S4 Table.

Next, delivery records for a person were assessed in order of occurrence. Each delivery record was compared to live births, stillbirths, ectopic pregnancies, abortions, and deliveries already classified as outcomes for a patient, and were retained if they occurred outside the required window(s) of time between outcomes from S3 Table. For example, if a patient had a delivery record more than 168 days after a live birth record classified in the first step and an abortion record classified in the fourth step occurred more than 70 days after the delivery then the delivery record would be retained as an outcome for the patient. Delivery outcomes classified in the current step were also considered if they occurred prior to the delivery record in consideration. If an antenatal visit or pregnancy confirmation record was found within 42 days of a delivery outcome classified above then that delivery outcome was not retained. Delivery outcomes were re-classified as live births.

### Start date estimation

In the second step, pregnancy episode start dates were estimated. In S4 Table the following outcome-specific term windows are defined:

- Maximum pregnancy term - amount of time to search back from the outcome for all pregnancy markers that can be used to estimate start date. This time period should be the longest possible gestation period for an outcome so as to capture early markers. For example, the maximum pregnancy terms for live births and ectopic pregnancies are 301 and 84 days respectively.
- Minimum pregnancy term - Shortest possible gestational period for each outcome. For example, the minimum pregnancy term for live births and ectopic pregnancies respectively are 161 and 42 days respectively.
- ‘Retry’ period: number of days a subsequent pregnancy episode is clinically possible following a preceding pregnancy outcome outcome; this time window indicates the minimum number of days after the outcome another pregnancy may be initiated. For example, another pregnancy episode cannot begin until 28 days after a live birth episode.

All time windows were determined based on estimates in the literature specific to each outcome and through clinical review. Pregnancy start markers were considered for an episode if they created a pregnancy term length that was greater than the minimum term length and less than the maximum term length from S4 Table, and did not precede the first occurring prior outcome date plus the retry period for that outcome.

A hierarchy of available pregnancy markers was chosen that reflects their potential accuracy to estimate start of the pregnancy episode. Pregnancy markers that provide gestational age directly such as last menstrual period, gestational age in weeks and fertility procedures that date conception were considered to be most accurate. Other types of markers considered were commonly administered pregnancy screening tests during narrow gestational age windows such as nuchal ultrasounds, markers that indicate possible first antenatal visit for the pregnancy such as amenorrhea, and average gestational age estimates.

In order to select and place pregnancy start markers in the hierarchy, start estimates from possible pregnancy markers were compared to start estimates from assisted conception procedures by pregnancy outcome in the Optum database. Among pregnancy episodes with assisted conception, the percentages of possible additional markers that were within two weeks prior or after the fertility procedure-based start date were calculated. This information was used to determine which markers were most proximal to the fertility procedure date in order to place them in the hierarchy for estimating starts in pregnancies without fertility procedures. The same analysis was done with nuchal ultrasounds substituted for assisted conception procedures. See S5 Table and S6 Table for results.

Pregnancy episode start dates were inferred by identifying the highest ranking observed marker from the pregnancy start marker hierarchy below. In other words, if a person had a last menstrual period date in their record that was used as the pregnancy episode start date. If no ‘last menstrual date’ was available, we looked for ‘gestational age record’, and if that was not available, we looked for a ‘fertility procedure’. If no pregnancy start markers were available to estimate pregnancy start, we used an outcome-specific estimate. After the highest ranking observed marker was identified the specified number of days was subtracted from the start marker date:

1. Last menstrual period date
2. Gestational age record date minus gestational age in days
3. Fertility procedure which dates conception record date minus 13 days
4. Nuchal ultrasound record date minus 89 days
5. Alpha fetoprotein test record date minus 123 days
6. Amenorrhea record date minus 55 days
7. Urine pregnancy record date minus 55 days
8. Outcome specific estimates (average gestational age estimate): If a premature term marker was associated with an episode (at least one premature marker between 1) largest of maximum term start date and prior outcome date plus retry period and 2) 30 days after outcome), the episode was classified as premature, otherwise it was considered to be full-term. From Table 5, the outcome specific gestational age estimate was chosen based on outcome and term and subtracted from the outcome date to obtain a pregnancy start date. For example, for a premature live birth outcome the gestational age estimate is 245 days, while term live birth gestational age estimate is 280 days. If the immediately prior outcome date plus days required for subsequent pregnancy initiation or ‘retry period’ was greater than the estimated start, then pregnancy start became the prior outcome date plus the retry period.

For nuchal ultrasound and alpha fetoprotein test screening tests, the midpoint (89 and 123 days respectively) of gestational age guidelines for administration from British Columbia was used to estimate the start of the pregnancy episode; midpoints were comparable in the US and UK. Amenorrhea and urine pregnancy start date estimations were estimated to be 55 days prior to the date of these records, based on the assumption that a woman’s first antenatal visit is generally 8-10 weeks after her last menstrual period date.

We adjusted the final pregnancy start date estimated with amenorrhea, urine pregnancy and average gestational age estimates with contraceptive drug exposure, pregnancy confirmation markers, antenatal GP visit, pregnancy complication, and threatened abortion records if present. These markers were considered for adjustment if they created a pregnancy term length that was greater than the minimum term length and less than the maximum term length from S4 Table, and did not precede the first occurring prior outcome date plus the retry period for that outcome. In the first adjustment step, if at least one contraceptive drug exposure was found after the estimated pregnancy start within the time window above, then the pregnancy estimated start was moved forward to the contraceptive drug exposure with the latest date. If there was no prior contraceptive exposure adjustment and one or more pregnancy confirmation, antenatal GP visit, pregnancy complication, or threatened abortion records were present within the time window mentioned above, then the pregnancy estimated start was moved back to the record in the classes above with the earliest date.

For nuchal ultrasound and alpha fetoprotein test screening tests, the midpoint (89 and 123 days respectively) of gestational age guidelines for administration from British Columbia was used to estimate the start of the pregnancy episode; midpoints were comparable in the US and UK. Amenorrhea and urine pregnancy start date estimations were estimated to be 55 days prior to the date of these records, based on the assumption that a woman’s first antenatal visit is generally 8-10 weeks after her last menstrual period date.

Pregnancy episodes were included in the final cohort if the patient was female, between 12 and 55 years of age (to include episodes amongst females with early menarche and mid-life fertility treatments) and had continuous enrollment during their pregnancy episode. Women were allowed to have multiple pregnancy episodes. Any outcome that did not have at least two associated pregnancy markers was excluded.
