# Supplementary material for: Inferring pregnancy episodes and outcomes within a network of observational databases
Source: PLoS One. 2018 Feb 1;13(2):e0192033. doi: 10.1371/journal.pone.0192033 (PMC5794136; doi:10.1371/journal.pone.0192033)
Supplement: S1 Fig — (DOCX) [file pone.0192033.s010.docx]

| **Pregnancy episode algorithm pseudocode: outcomes assessment** |
| --- |
| --Determine if a pregnancy outcome event is valid  --(evt:potential outcome, Outcomes: prior classified outcomes, pregnancyEvents: all pregnancy events for a person)  **declare** isOutcome(evt, Outcomes, pregnancyEvents)  {*--* *Outcome Types****:*** *LB: Livebirth, SB: Stillbirth, ECT: Ectopic Pregnancy, AB: Spontaneous and induced abortion, DELIV: Delivery*  *-- invalid windows are spans of time around prior valid outcomes where a new outcome cannot occur: see section 2.1.2*  **if** (evt within any invalid window) **then**     **return** false;  *-- For outcomes 'SB, ECT, AB, DELIV: no antenatal or pregnancy confirmation within 42 days of outcome date*  **if** evt.type **in** {'SB, ECT, AB, DELIV'}  **and** **exists**(x **in** pregnancyEvents **where** x within 42 days after evt **and** x.type **in** {'PCONF', 'AGP'}) **then**     **return** false;  *-- ECT_TX:ectopic pregnancy-related procedure or methotrexate exposure or a ECT_COMORBID:concept identified as highly*  *associated with ectopic pregnancy by a disproportionality analysis*  **if** evt.type **in** {‘ECT’} **and**  **not** **exists**(x **in** pregnancyEvents **where** x within 14 days after evt **and** x.type in {'ECT_TX',’ECT_COMORBID}) **then**     **return** false;  **return** true;} *-- no invalid conditions found, so return true*  --*Begin algorithm, Input: All pregnancy events sorted by date, Output: Validated pregnancy outcome type and date*  **set** Outcomes={} --*Set current valid pregnancy outcome set to empty set*  **for each** person p  **set** personPe={pregnancy records for person p from all pregnancy events}  **for each** ot **in** {‘LB’,’SB’,’ECT’,’AB’,’DELIV’}  **set** typePe={x **in** personPe **where** x.type **is** ot}  **for** **each** typePe_i **in** typePe  *-- Reassign abortion outcome date to last abortion date within 2 weeks after*  **if** (ot == ‘AB’) **then**  **set** abortions={x **in** personPe **where** x.type **in** {‘AB’} **and** x within 2 wks after typePe_i }  **if** abortions **is not empty then**  typePe_i.event_date=**max**(abortions).event_date --*Reassign to latest abortion date*  **end if**  **end if**  *-- Reassign ectopic pregnancy outcome date to last treatment date within 2 weeks after*  **if** (ot == ‘ECT’) **then**  **set** ectopics={x **in** pe **where** type(x) **in** {‘ECT_TX} **and** x within 2 wks after temp_pe_i}  **if** ectopics **is not empty then**  typePe_i.event_date=**max**(ectopics).event_date --*Reassign to latest ectopic treatment date*  **end if**  **end if**  **If** isOutcome { typePe_i, Outcomes**,** personPe **} then**  Outcomes = Outcomes **union** typePe_i  **end for**  **end for**  **end for**  **--**reassign DELIV to LB outcome |
