# Supplementary material for: Inferring pregnancy episodes and outcomes within a network of observational databases
Source: PLoS One. 2018 Feb 1;13(2):e0192033. doi: 10.1371/journal.pone.0192033 (PMC5794136; doi:10.1371/journal.pone.0192033)
Supplement: S2 Fig — (DOCX) [file pone.0192033.s011.docx]

| **Pregnancy episode algorithm pseudocode: episode start estimation** |
| --- |
| *--Adjust estimated starts based on contraception and pregnancy confirmation markers within start window for each outcome*  *--Retry period=time period needed for initiation of 2^nd^ pregnancy for each possible outcome*  *--Start window=greater of (max possible term start, prior outcome date + retry period) to min possible term start*  --*Premature marker window= largest of (max possible term start, prior outcome date + retry period) to 30 days after outcome*  **declare** adjustStart(episodeStart,pregnancyEvents)  {**if** **exists**(any x **in** pregnancyEvents **where** x within start window **and** x.type **in** {'CONTRACEPTION'}) **and**  episodeStart<max(set of all x.event_date) **then** **set** episodeStart=max(set of all x.event_date)  **else if exists** (any x **in** pregnancyEvents **where** x within start window **and** x.type **in** {'PCONF'}) **and**  episodeStart>min(set of all x.event_date)  **then** **set** episodeStart=min(set of all x.event_date)  **else** episodeStart=episodeStart;}  **set** pregnancyEpisodes = {} --*will contain final pregnancy episodes*  **set** priorOutcome={closest prior Validatedoutcomes for each outcome}  **for each** outcome in validatedOutcomes  -- *Pregnancy episode start marker types****:*** *LMP: Last menstrual period date, GEST: Gestational age record, FERT: Assisted*  *conception procedure date, ULS: Nuchal ultrasound date, AFP: Alpha feto protein test date, AMEN: Amenorrhea record date,*  *URINE: Urine pregnancy test date*  **if** exists(any st **in** pregnancyEvents where st.type == ‘LMP’ **and** max(set of all st.event_date) within start window)  **then set** episodeStart=max(set of all st.event_date)  **else if** exists(any st **in** pregnancyEvents where st.type == ‘GEST’ **and** max(set of all st.event_date-(gestational weeks*7+1))  within start window) **then set** episodeStart=max(st.event_date)-(gestational weeks*7+1)  **else if** exists(any st **in** pregnancyEvents where st.type == ‘FERT’ **and** min(set of all st.event_date)-13 within start window)  **then set** episodeStart= min(set of all st.event_date)-13  **else if** exists(any st **in** pregnancyEvents where st.type == ‘ULS’ **and** min(set of all st.event_date)-89 within start window)  **then set** episodeStart= min(set of all st.event_date)-89  **else if** exists(any st **in** pregnancyEvents where st.type == ‘AFP’ **and** min(set of all st.event_date)-123 within start window)  **then set** episodeStart= min(set of all st.event_date)-123  **else if** exists(any st **in** pregnancyEvents where st.type in (‘AMEN’,’URINE) and min(set of all st.event_date)-55 within start  window) **then set** episodeStart= adjustStart(min(set of all st.event_date)-55, pregnancyEvents)  --default cases  **else if** outcome.type==’LB’  **and** not (**if** **exists**(any x **in** pregnancyEvents **where** x within premature marker window **and** x.type **in** {'PREM'}))  **then set** episodeStart=  adjustStart(max(outcome.event_date – 280 , priorOutcome.event_date+retry period), pregnancyEvents)  **else if** outcome.type==’LB’  **and** not (**if** **exists**(any x **in** pregnancyEvents **where** x within premature marker window **and** x.type **in** {'PREM'}))  **then set** episodeStart=  adjustStart(max(outcome.event_date – 245 , priorOutcome.event_date+retry period), pregnancyEvents)  **else if** outcome.type==’SB’  **then set** episodeStart=  adjustStart(max(outcome.event_date – 196 , priorOutcome.event_date+retry period), pregnancyEvents)  **else if** outcome.type==’ECT’  **then set** episodeStart=  adjustStart(max(outcome.event_date – 56 , priorOutcome.event_date+retry period, pregnancyEvents)  **else if** outcome.type==’AB’  **then set** episodeStart=  adjustStart(max(outcome.event_date – 70 , priorOutcome.event_date+retry period, pregnancyEvents)  pregnancyEpisodes = pregnancyEpisodes **union** **new** pregnancyEpisode(episodeStart, outcomeEvent_date,outcomeEvent.type)  **end for** |
