# Supplementary material for: Inferring pregnancy episodes and outcomes within a network of observational databases
Source: PLoS One. 2018 Feb 1;13(2):e0192033. doi: 10.1371/journal.pone.0192033 (PMC5794136; doi:10.1371/journal.pone.0192033)

### Illustrative Patient Example

S3 Fig contains an illustration of the steps performed by the pregnancy episode algorithm for a sample patient resulting in three classified pregnancy episodes and estimated start dates. In this example, three pregnancy episodes are classified for the patient by the algorithm 1) live birth: day 0-280 2) ectopic pregnancy: day 435-510 3) stillbirth episode: day 524-720. The figure identifies nine sequential steps detailed below that the algorithm would perform in order to classify the three pregnancy episodes.

Pregnancy markers detailed in the legend are arranged on a timeline in S3 Fig. The operations illustrated include outcome classification (circle steps 1-3 and diamond steps 1-3) and start date estimation (diamond steps 4-5). Outcomes that are classified by the algorithm are represented by black solid shapes, while unclassified outcomes (not included as an outcome for the patient) are represented by white shapes with broken line borders. Pregnancy markers used to classify outcomes such as methotrexate treatment are also represented by shaded shapes. Pregnancy start markers are represented on the timeline below the potential outcome records. Black doughnuts represent the start markers highest in the start hierarchy that were chosen to infer start. White doughnuts represent start markers that were not utilized by the algorithm.

The following steps are described in order of execution by the algorithm:

1. Circle step 1: a live birth outcome is classified
2. Diamond step 2: a second live birth is unclassified because it occurs too close to a prior live birth
3. Circle step 2: a stillbirth outcome is classified
4. Circle step 3: an ectopic pregnancy is classified
5. Diamond step 3: ectopic pregnancy outcome date is changed to methotrexate treatment date within 14 day period after initial ectopic pregnancy diagnosis
6. Diamond step 1: Abortion record is unclassified due to pregnancy confirmation record within 42 days after initial abortion record
7. Diamond step 4: infer pregnancy start date for live birth identified in circle step 1; start=nuchal ultrasound procedure minus 89 days (day 0 on the timeline)
8. Diamond step 5: infer pregnancy start date for ectopic pregnancy; start=Amenorrhea record minus 55 days (day 435 on the timeline)
9. Estimated gestational age default chosen for stillbirth record identified in circle step 2 (day 524 on the timeline)

**S3 Fig Illustration of steps performed by pregnancy episode algorithm for sample patient with three classified pregnancy episodes and estimated start dates.**

Legend Start markers: LMP: Last menstrual period date; GEST: Gestational age record; FERT: Fertility procedure; ULS: Nuchal ultrasound; AFP: Alpha-fetoprotein; AMEN: Amenorrhea record; Urine: Urine pregnancy test; Default: Gestational age estimate.


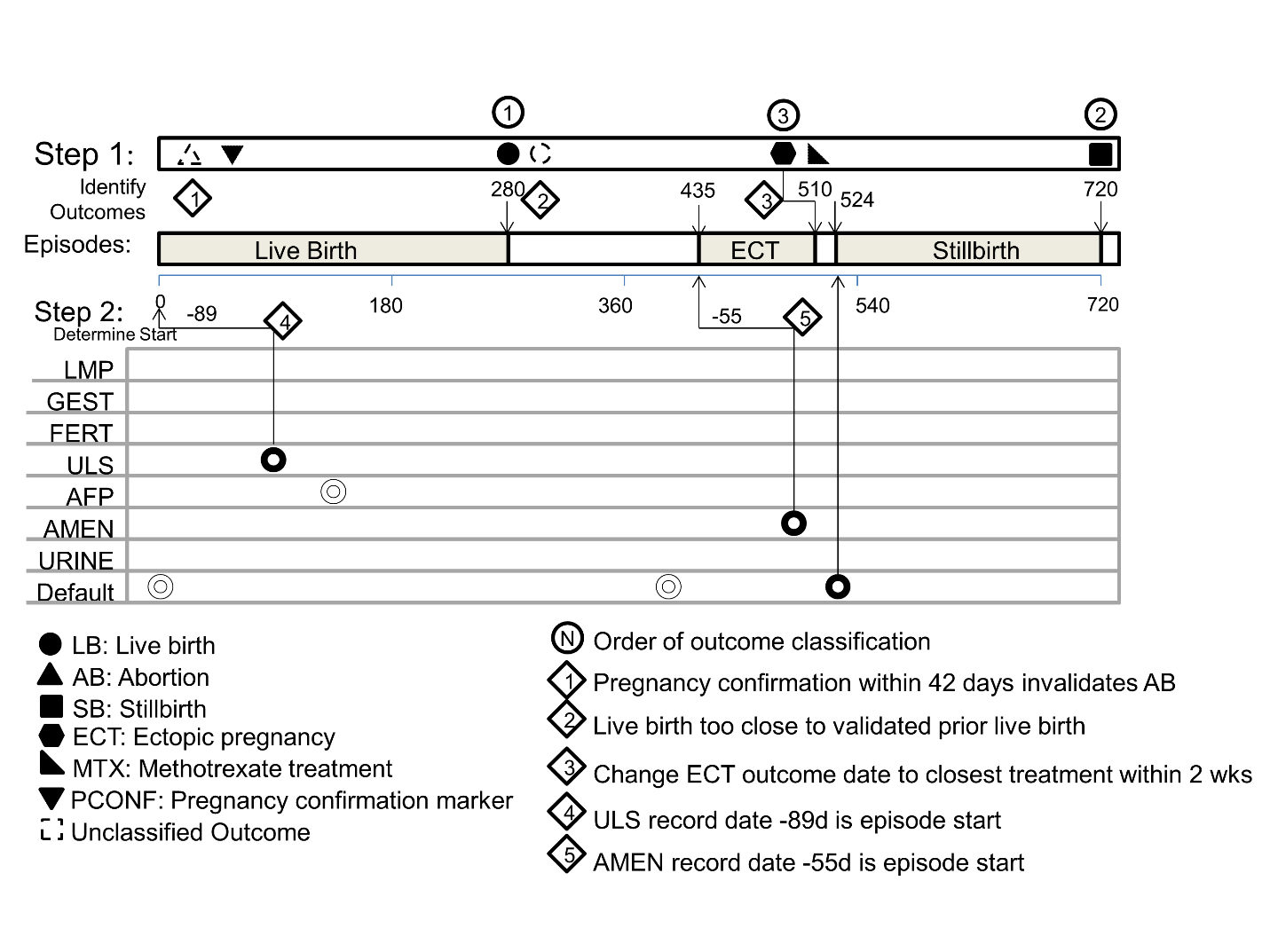

Supplement: S3 Fig — (DOCX) [file pone.0192033.s012.docx]
