# Supplementary material for: Inferring pregnancy episodes and outcomes within a network of observational databases
Source: PLoS One. 2018 Feb 1;13(2):e0192033. doi: 10.1371/journal.pone.0192033 (PMC5794136; doi:10.1371/journal.pone.0192033)
Supplement: S1 ISAC — (DOCX) [file pone.0192033.s018.docx]

|  |
| --- |
| **Janssen Research & Development*** |
|  |
| **Epidemiology Study Protocol** |
|  |
| **An algorithm to determine pregnancy episodes and outcomes in Janssen CDM databases** |

*Janssen Research & Development (JRD) is a global organization that operates through different legal entities in various countries. Therefore, the legal entity acting as the sponsor for studies of Janssen Research & Development may vary. The term "sponsor" is used throughout the protocol to represent these various legal entities; the sponsor is identified on the Contact Information page that accompanies the protocol.

**Issue/Report Date:** 06 March 2015

**Prepared by:** Janssen Research and Development

**Document No.:** EDMS‑XXXX‑XXXXXXX:2.0

**Confidentiality Statement**

The information in this document contains trade secrets and commercial information that are privileged or confidential and may not be disclosed unless such disclosure is required by applicable law or regulations. In any event, persons to whom the information is disclosed must be informed that the information is *privileged* or *confidential* and may not be further disclosed by them. These restrictions on disclosure will apply equally to *all* future information supplied to you that is indicated as *privileged* or *confidential*.

AUTHORS

Amy Matcho, Manager, Epidemiology Analytics, Epidemiology, Janssen Research and Development

Patrick Ryan, PhD, Sr. Director, Epidemiology, Janssen Research and Development

Daniel Fife, MD, Sr. Director, Epidemiology, Janssen Research and Development

Andrew Friedman, MD, Sr. Director Global Labeling, GRA, Janssen Research and Development

Dina Gifkins, PhD, Associate Director, Epidemiology, Janssen Research and Development

Chris Knoll, Manager, Epidemiology Analytics, Epidemiology, Janssen Research and Development

note

The outline of this template is consistent with the Guidelines for Good Pharmacoepidemiology Practices (GPP) and The Strengthening the Reporting of Observational Studies in Epidemiology (STROBE) checklist.

TABLE OF CONTENTS

SYNOPSIS 4

1. INTRODUCTION 6

1.1. Background 6

1.2. Overall Rationale for the Study 7

2. STUDY OBJECTIVES 7

2.1. Primary Objective(s) 7

2.2. Secondary Objective(s) 7

3. OVERVIEW OF STUDY DESIGN 7

3.1. Study Design 7

3.2. Study Design Rationale 7

4. STUDY POPULATION 7

4.1. Patient Selection 7

4.1.1. Inclusion Criteria 8

4.1.2. Exclusion Criteria 8

4.1.3. Data Source(s) 8

5. STATISTICAL METHODS 9

5.1. Sample Size and Study Power 9

5.2. Measurement 9

5.2.1. Exposure Definition and Measures 9

5.2.2. Outcome Definition and Measures 9

5.2.2.1. Concept Code List 9

5.2.2.2. Summary of Pregnancy Episode Algorithm 10

5.2.2.2.1. 1st Step in the Pregnancy Episode Algorithm: Assignment of Outcomes 10

5.2.2.2.2. 2nd Step in the Pregnancy Episode Algorithm: Assignment of Start Dates 13

5.2.3. Potential Confounders and Effect Modifiers 16

5.3. Analyses 16

5.3.1. Specific Comparisons 16

5.4. Missing Data Handling 17

5.5. Data Preparation 17

5.6. Statistical Analysis Plan 17

5.6.1. Descriptive Analyses 17

5.6.2. Quantitative Analyses 17

5.6.3. Sensitivity and Other Supplementary Analyses 17

5.7. Data Quality Assurance 17

5.7.1. Validation Procedures 17

6. STUDY LIMITATIONS 18

7. ETHICAL ASPECTS 18

7.1. Privacy of Personal Data 18

# SYNOPSIS

**LAY SUMMARY**

Administrative claims databases and electronic health records (EHR) are valuable resources for pregnancy research since pregnant women are excluded from randomized clinical trials (RCTs). In general, direct measures of gestational age are not available in claims databases and EHRs and an algorithm based on data available must be developed and validated in order to define the entire pregnancy episode from start to pregnancy outcome.

Our aim is to develop an algorithm to define pregnancy episodes for a network of population-based databases using the OMOP Common Data Model (CDM). We will attempt to identify all possible pregnancy outcomes with the algorithm, including live birth (at least one), stillbirth, delivery record only, trophoblastic disease, ectopic pregnancy, induced abortion and spontaneous abortion. Pregnancy episode start dates will be inferred with various pregnancy markers such as last menstrual period and nuchal ultrasound dates.

Our publication strategy will involve disseminating the algorithm methods and results from the electronic profile validation through a journal which focuses on epidemiology and/or drug safety. The validation should provide insight into the accuracy of various operating characteristics of the algorithm, such as start date inferral and outcome assignment. Validation results will be presented separately for each database in one publication in order to highlight the fact that it was possible to use one algorithm for all databases converted to the CDM at Janssen.

INTRODUCTION

Administrative claims databases and electronic health records (EHR) are valuable resources for pregnancy research since pregnant women are excluded from randomized clinical trials (RCTs). In general, direct measures of gestational age are not available in claims databases and EHRs and an algorithm based on data available must be developed and validated in order to define the entire pregnancy episode from start to pregnancy outcome.

A study has not been performed that has attempted to accurately define pregnancy episodes including pregnancy start date on a network of population-based databases from the US and the UK using the OMOP Common Data Model (CDM). Using the CDM allows us to create one set of pregnancy concepts and one algorithm that can be applied to all data sources converted to the CDM, despite disparate native source code dictionaries and data structures found within each database. Additionally, prior observational data studies in which pregnancy episodes were identified have either been based upon populations that were potentially not generalizable or have only examined live birth outcomes. It is important to examine all possible outcomes (e.g. spontaneous and induced abortion, stillbirth, ectopic pregnancies etc.) as patient characteristics, prescribing patterns etc. in these populations may be dissimilar from patterns in pregnancies ending in live births.

OBJECTIVES

Our aim is to define pregnancy episodes and their outcomes (all possible outcomes including live births, stillborn, spontaneous and induced abortion, ectopic pregnancies etc.) within 3 US-based administrative claims databases: Truven MarketScan Commercial Claims and Encounters (CCAE), Truven MarketScan Multi-state Medicaid (MDCD), and the Optum ClinFormatics (Optum) database and the Clinical Practice Research Datalink (CPRD) which is a UK based general practice electronic health record.

These will be validated with review of 50 CPRD and 50 Optum randomly drawn pregnancy episodes from each outcome (6 outcomes X 50 X 2) for a total of 600 profiles by clinical personnel. Proportion of episodes by outcome which refer to an actual pregnancy, proportion of episodes by outcome with correct outcome, and proportion of episodes by outcome with correct start date (or plausible start date if inferred) for each database will be determined.

OVERVIEW OF STUDY DESIGN

This study follows a retrospective cohort design. 7 pregnancy episode outcome cohorts will be created and characterized from women with any pregnancy markers or outcomes:

- Live birth (at least one)
- Stillbirth
- Delivery-Live birth or stillbirth
- Trophoblastic Disease
- Ectopic pregnancy
- Induced abortion
- Spontaneous abortion

Women are allowed multiple pregnancy episodes.

STUDY POPULATION

Women between 12 and 55 years of age who were enrolled continuously during their pregnancy episode defined by the algorithm within the 4 databases with any pregnancy markers or outcomes.

STATISTICAL METHODS

For each pregnancy episode, we will determine the beginning and end of the pregnancy episode and determine the outcome.

ABBREVIATIONS

| IRB | Institutional Review Board |
| --- | --- |
| MedDRA | Medical Dictionary for Regulatory Activities |
| CCAE | Truven MarketScan Commerical Claims and Encounters |
| MDCR | Truven MarketScan Medicare Supplemental Beneficiaries |
| MDCD | Truven MarketScan Multi-state Medicaid |
| Optum | Optum ClinFormatics |
| OMOP | Observational Medical Outcomes Partnership |
| CDM | Common Data Model |
| CI | Confidence interval |
| CPRD | Clinical Practice Research Datalink |

## INTRODUCTION

### Background

Administrative claims databases and electronic health records (EHR) are valuable resources for pregnancy research since pregnant women are excluded from randomized clinical trials (RCTs). In general, direct measures of gestational age are not available in claims databases and EHRs and an algorithm based on data available must be developed and validated in order to define the entire pregnancy episode from start to pregnancy outcome.

Many algorithms have been documented in the literature. Hornbrook et al. created an algorithm to identify pregnancy episodes and outcomes from Kaiser Permanente Northwest automated US healthcare records [[1](#_ENREF_1)]. Beginning and end of the pregnancy episode along with a range of outcomes including live birth, spontaneous and induced abortion, stillbirth etc. were determined for each pregnancy using condition and procedure codes from outpatient and inpatient data, laboratory tests and pharmacy records. In their electronic health data, gestational age was available for some pregnancies in hospital discharge data, otherwise gestational age of the infant was estimated using national median gestational age by outcome type. Percent agreement between their algorithm and medical records review ranged from 91-98% for outcome, gestational age and outcome date. With their algorithm, 68% of all pregnancy episodes defined ended in a live birth, 17% ended in induced abortion, 13% ended in spontaneous abortion, 1% ended in ectopic pregnancy and <1% ended in stillbirth, trophoblastic pregnancy, live or stillbirth or unknown outcomes.

Margulis et al. determined with a population-based cohort of mother-child pairs in British Columbia that subtracting 245 days from live births with pre-term delivery ICD-9 codes and 273 days for those without provided best estimates of the beginning of pregnancy as compared to the hospital discharge record (68.1% of estimated gestational age at birth were within 1 week of the hospital discharge estimate) [[2](#_ENREF_2)]. A second algorithm in this effort attempted to determine beginning of pregnancy with screening test claims such as alpha fetoprotein in live births usually administered within narrow gestational-age windows (45.4% of estimated gestational age at birth were within 1 week of the hospital discharge estimate).

Two UK-based studies involving CPRD or The Health Improvement Network (THIN) which are both drawn from general practice data in the UK defined pregnancy episodes by determining the appropriate end of pregnancy marker and mapping it to the first found early pregnancy marker [[3](#_ENREF_3), [4](#_ENREF_4)]. A more recent study utilizing THIN data used last menstrual period (LMP) date which was present for 50.8% of pregnancies to determine beginning of pregnancy [[5](#_ENREF_5)].

A study has not been performed that has attempted to accurately define pregnancy episodes including pregnancy start date on a network of population-based databases from the US and the UK using the OMOP Common Data Model. Using the CDM allows us to create one set of pregnancy concepts and one algorithm that can be applied to all data sources converted to the CDM, despite disparate native source code dictionaries and data structures found within each database.

Additionally, prior observational data studies examining pregnancy using the types of algorithms described above have either been based upon populations that were not generalizable or have only examined live birth outcomes [[6-8](#_ENREF_6)]. It is important to examine all possible outcomes (e.g. spontaneous and induced abortion, stillbirth, ectopic pregnancies etc.) as patient characteristics, prescribing patterns etc. in these populations may be dissimilar from patterns in pregnancies ending in live births.

### Overall Rationale for the Study

## STUDY OBJECTIVES

### Primary Objective(s)

Our primary objective is to evaluate an algorithm that will determine pregnancy episodes (pregnancy start to end) and outcomes for the episodes (including outcomes other than live birth) in all applicable Janssen network databases.

### Secondary Objective(s)

Hypothesis:

This pregnancy study is descriptive in nature and does not involve any statistical tests.

## OVERVIEW OF STUDY DESIGN

### Study Design

This study follows a retrospective cohort design. 7 pregnancy episode outcome cohorts will be created and characterized from women with any pregnancy markers or outcomes:

- Live birth (at least one)
- Stillbirth
- Delivery-Live birth or stillbirth
- Trophoblastic Disease
- Ectopic pregnancy
- Induced abortion
- Spontaneous abortion

Women are allowed multiple pregnancy episodes and the unit of analysis will be the pregnancy episode. Valid pregnancy outcomes for each patient will be chosen using an algorithm that utilizes an outcome assessment hierarchy, i.e. live births are assessed for validity first for each patient, any that are too close to another live birth are discarded. Still births for the same patient are then assessed to see if they are too close to all prior assessed live births and any other valid still births etc. Start dates will be assigned using pregnancy markers in a hierarchical fashion also (last menstrual period date records considered as start of pregnancy first, next considered are gestational age estimate records, etc.) See page 12 for a full description of the algorithm.

In medical practices, pregnancy episodes traditionally begin with the last menstrual period date and end with the birth outcome date as defined by the algorithm. The first trimester is defined as week 1-13, 2^nd^ trimester week 14-26 and 3^rd^ trimester week 27-40. For our episodes, the first two weeks of the first trimester will be defined as a pre-exposure period as conception most likely does not happen prior to this time.

### Study Design Rationale

A retrospective cohort design will allow pregnancies to be identified at pregnancy start as defined above and followed longitudinally to the birth outcome.

## STUDY POPULATION

### Patient Selection

NOTE: All subject data for this study are being collected using databases only.

This analysis will be conducted within 3 US-based administrative claims databases: Truven MarketScan Commercial Claims and Encounters (CCAE) which provides data from 1/2000-10/2014, Truven MarketScan Multi-state Medicaid (MDCD) (data available from 1/2006-12/2013), and the Optum ClinFormatics (Optum) database (data available from 10/2005-9/2014). The analysis will also be conducted within the Clinical Practice Research Datalink (CPRD), which is a UK based general practice electronic health record which provides data from 9/1987-7/2014. All databases are transformed to the OMOP Common Data Model (CDM) v4. Details about the OMOP CDM are available at: <http://omop.org/CDM>. One standard analysis program will be developed for this protocol, and executed consistently across all databases using the OMOP CDM.

The transformation of CPRD data into the OMOP CDM V4 and the validation performed at Janssen has been described previously [[9](#_ENREF_9)]. Validation of the conversion of Read codes classified as conditions to SNOMED-CT results from the above publication are as follows: “99.9% of condition records in the CDM condition occurrence file and 98.9% of condition terms were mapped to the SNOMED-CT dictionary. The top 100 occurring conditions in the CPRD CDM made up 47% of the condition data and all were mapped and classified correctly. 97.7% of procedure records in the procedure occurrence file and 86.4% of procedure terms were mapped to the SNOMED-CT dictionary. The top 100 occurring procedures in the CPRD CDM made up 68.5% of the procedure data and all were mapped and classified correctly. The final observation file with all 3 observation record sources (lifestyle/clinical, lab and Read code observation data) had 94.2% of observations records and 75.2% of observation terms mapped to either the LOINC or SNOMED-CT dictionaries. The top 100 occurring observations in the CPRD CDM made up 60% of the observation data and all were mapped and classified correctly except four unmappable codes from the lifestyle/clinical data.” Also, prevalences for selected conditions across CPRD raw and CDM data were equivalent and **r**esults between the replication raw data and CDM study agreed for conditions, demographics and lifestyle data. [[9](#_ENREF_9)]. Additionally, a commentary piece by Rijnbeek describes advantages and potential limitations of the CPRD CDM conversion performed by Matcho et al [[10](#_ENREF_10)].

All data available within all data sources will be considered for use for construction of the pregnancy episodes. All analyses are retrospective based on existing de-identified patient-level data, and will not require prospective data capture or patient re-identification for source record verification or other purposes. Cohorts will be constructed from inclusion criteria specified below.

#### Inclusion Criteria

Women between 12 and 55 years of age who were enrolled continuously during their pregnancy episode determined from pregnancy markers or outcomes within the 4 databases will be included in the study. This age group was chosen to detect pregnancies amongst girls with earlier menarche as well as pregnancies in older women (primarily due to extension of fertility with medical interventions) [[1](#_ENREF_1)].

#### Exclusion Criteria

Delete episodes which end after or start prior to the patient’s valid observation period.

#### Data Source(s)

CCAE is an administrative health claims database for active employees, early retirees, COBRA continues, and their dependents insured by employer-sponsored plans (individuals in plans or product lines with fee-for-service plans and fully capitated or partially capitated plans). CCAE captures person-specific clinical utilization, expenditures, and enrollment across inpatient, outpatient, prescription drug, and carve-out services. It also includes results for outpatient lab tests processed by large national lab vendors. ~108 million patients are available in CCAE from 1/2000-10/2013.

MDCD contains the pooled healthcare experience Medicaid enrollees from multiple states. It includes inpatient services and prescription drug claims, as well as information on enrollment, long-term care, and other medical care. MDCD has ~16 million lives from 1/2006-12/2012.

Optum represents an administrative health claims database made mostly of members of United Healthcare, who are enrolled in commercial plans, Medicaid and Legacy Medicare Choice with both medical and prescription drug coverage. Optum captures person-specific clinical utilization, expenditures, and enrollment across inpatient, outpatient, prescription drug, and carve-out services. It also includes results for outpatient lab tests processed by large national lab vendors. Optum has ~36 million lives from 1/2006-12/2012.

CPRD contains anonymised longitudinal electronic health records from primary care practices in UK from 1987-present day. It is a patient management system with many aspects of patient care covered, including diagnoses, prescriptions, signs and symptoms, procedures, labs, lifestyle factors, clinical and administrative/social data. ~11.5 million patients are available in the database.

All databases are transformed to the OMOP Common Data Model (CDM) v4. Details about the OMOP CDM are available at: <http://omop.org/CDM>. One standard analysis program will be developed for this protocol, and executed consistently across all databases using the OMOP CDM.

## STATISTICAL METHODS

### Sample Size and Study Power

All qualifying patients will be included within each cohort for this descriptive summary. No statistical tests are being performed, so no sample size or study power calculations are required.

### Measurement

#### Exposure Definition and Measures

No exposures defined or measured for this protocol.

#### Outcome Definition and Measures

##### Concept Code List

One code set (used to gather patient pregnancy records) will be utilized for all data sources in order to determine pregnancy outcomes and markers, as the OMOP CDM allows for standardization of clinical content across all databases. All content (conditions, procedures and observations) in the OMOP CDM are referred to by concepts. The OMOP Standard Vocabularies are used to understand and make use of these concepts. Native source codes are mapped to the dictionary that is considered standard for that domain (conditions, procedures, etc.) in the Standard Vocabularies. For instance, Read condition codes from CPRD and ICD-9 condition codes from US claims databases are mapped to SNOMED-CT concepts in all of our CDM versions of the 4 databases. SNOMED_CT is the standard dictionary for conditions and LOINC is the standard dictionary for labs and observations in the CDM. There is no standard dictionary for procedures. Procedures will be identified by ICD-9 procedure, CPT-4, HCPCS and SNOMED-CT concepts.

Our pregnancy concept list was developed utilizing the OMOP Standard Vocabularies. SNOMED-CT concepts indicating pregnancy care, screening, outcomes etc. were identified and all descendants were gathered into one code list. Read and ICD-9 pregnancy chapter searches identified concepts not picked up by the SNOMED-CT concept search as the range and terminologies of pregnancy-related concepts are broad and some pregnancy concepts are descendants of non-pregnancy higher-level concepts. Hornbrook et al. [[1](#_ENREF_1)] shared the source code list used in their analysis using a US administrative claims data source and we also validated our concepts against their source codes.

The categorized pregnancy concepts that will be used to create the pregnancy episodes are specified below. At this time concepts that had 100 or more records in the data were categorized. Categorizing the remaining source codes and validation will improve the accuracy of the pregnancy episode algorithm.

The pregnancy outcomes will be defined with concepts from these categories (excepting premature, post-term and full-term concepts which will be used to infer pregnancy start dates):

| Premature | PREM |
| --- | --- |
| Post-term | POSTT |
| Full-term | FT |
| Live Birth (in multiple births at least one live birth) | LB |
| Stillbirth | SB |
| Induced or legal abortion | AB |
| Delivery-Live birth or stillbirth | DELIV |
| Spontaneous abortion | SA |
| Ectopic pregnancy | ECT |
| Trophoblastic disease | TRO |

Pregnancy markers at various stages of the pregnancy episode include these categories:

| Confirmation of pregnancy | PCONF |
| --- | --- |
| Antenatal GP visits | AGP |
| Pregnancy complication codes | PCOMP |
| Labor and pre-delivery codes | LDEL |
| Threatened abortion | TA |
| Postpartum | POST |

Screening tests include concepts from these categories:

| Alpha fetoprotein screening tests | AFP |
| --- | --- |
| Amniocentesis screening tests | AMNIO |
| Obstetrical ultrasound | ULS |
| Gestational Diabetes screening | DIAB |
| Other lab tests or procedures indicative of pregnancy | OTEST |

Calculations involving recordings of ‘last menstrual period’ or gestational age will use concepts from these categories:

| Gestational age indicated | GEST |
| --- | --- |
| LMP | LMP |

##### Summary of Pregnancy Episode Algorithm

The algorithm to determine pregnancy episodes will include several steps. In the first step, all pregnancy outcomes for a patient will be assigned. In the second step, pregnancy start dates will be assigned.

###### 1st Step in the Pregnancy Episode Algorithm: Assignment of Outcomes

An outcome assessment hierarchy rational was chosen similar to that used in a study by Mikolajcyzk et al. Births unambiguously indicate the outcome took place, however spontaneous abortions and ectopic pregnancies may only indicate suspicion of occurrence of the event so these will be assessed after birth outcomes [[11](#_ENREF_11)]. Valid outcomes will be assessed in this order:

- Live birth (at least one)
- Stillbirth
- Delivery-Live birth or stillbirth
- Trophoblastic Disease
- Ectopic pregnancy
- Induced abortion
- Spontaneous abortion

Before searching for outcomes, remove all patients from consideration who only have gestational diabetes screening (DIAB) records without any other pregnancy categories, as urine and serum glucose tests have low specificity for pregnancy.

Outcomes will be found in the CDM condition_occurrence, procedure_occurrence and observation files. In addition to SNOMED-CT and procedure concepts in the CPRD CDM:

- live births can be indicated by LOINC concepts when the value_as_string field in the observation table=’Live birth’ or ‘Neonatal death’
- stillbirths can be indicated by LOINC concepts when the value_as_string field in the observation table=’Still birth’ or ‘Intra-partum death’
- deliveries can be indicated by LOINC concepts when the value_as_string field in the observation table=’Unknown’

The following steps assign the valid pregnancy outcomes:

1. Valid live birth (LB) outcomes will be assessed first. Starting with the first LB outcome (assumed to be true), the 2^nd^ LB outcome will be assessed to see if it occurs after the necessary time interval (see Table 1). Table 1 contains time windows that are based on the least amount of time a 2^nd^ outcome can happen after the 1^st.^  These can change based upon the specific outcomes under consideration. Minimum time allowed between two LB outcomes is 182 days. These time windows were adapted from the Hornbrook et al.[[1](#_ENREF_1)] algorithm and validated by in-house medical experts. If the 2^nd^ LB outcome happens too soon after the 1^st^, it will be removed from consideration and the 3^rd^ LB outcome will be compared to the 1^st^ LB outcome in the same manner. If the 2^nd^ LB outcome occurs after the time window allowed it will be classified as a valid outcome, and the 3^rd^ LB outcome will be compared to it; this process will be repeated until all LB outcomes for a patient are assessed.
2. Next, all stillbirth (SB) outcomes will be assessed. The first SB outcome found for a patient will be compared to all LB outcomes determined to be valid for the patient in the prior step. If all LB outcomes prior to the SB outcome occur 168 days prior to the SB outcome and all LB outcomes after the SB outcome occur 182 days after the SB outcome then the first SB outcome will be added to the valid outcome set. Each subsequent SB outcome will be assessed for validity in this manner against all LB and SB outcomes that have been determined to be valid using Table 1 in the prior step. In other words, if all outcomes prior to the subsequent SB outcome are prior to the minimum number of days for that outcome combination in Table 1 and all outcomes after the subsequent SB outcome are after the minimum number of days for that outcome in Table1 then the subsequent SB outcome will be added to the outcome set.
3. Next, all delivery (DELIV) outcomes will be assessed against all valid LB, SB and DELIV outcomes in the same manner as described above for SB and added to the valid outcome set if Table 1 criteria is met.
4. Next, ectopic pregnancy (TRO) outcomes will be assessed against all valid LB, SB, DELIV, and TRO outcomes in the same manner as described above for SB and added to the valid outcome set if Table 1 criteria is met. In order to eliminate TRO rule-out diagnoses, if there is an antenatal GP visit, pregnancy confirmation or pregnancy complication record within 42 days of the TRO record that TRO outcome will not be added to the valid outcome set.
5. Next, ectopic pregnancy (ECT) outcomes will be assessed against all valid LB, SB, DELIV, TRO and ECT outcomes in the same manner as described above for SB and added to the valid outcome set if Table 1 criteria is met. In order to eliminate ECT rule-out diagnoses, if there is an antenatal GP visit, pregnancy confirmation or pregnancy complication record within 42 days of the ECT record that ECT outcome will not be added to the valid outcome set.
6. Next, induced abortion (AB) outcomes will be assessed against all valid LB, SB, DELIV, TRO, ECT and AB outcomes in the same manner as described above for SB and added to the valid outcome set if Table 1 criteria is met.
7. Next, spontaneous abortion (SA) outcomes will be assessed against all valid LB, SB, DELIV, TRO, ECT, AB, and SA outcomes in the same manner as described above for SB and added to the valid outcome set if Table 1 criteria is met. In order to eliminate SA rule-out diagnoses, if there is an antenatal GP visit, pregnancy confirmation or pregnancy complication record within 42 days of the SA record that ECT outcome will not be added to the valid outcome set.

| Table 1: Minimum Number of Days Required to Identify Separate Pregnancy Outcomes | | |
| --- | --- | --- |
| First Pregnancy Outcome | Second Pregnancy Outcome | Minimum Days Between Outcomes |
| LB | LB | 182 |
| LB | SB | 168 |
| LB | DELIV | 168 |
| LB | TRO | 70 |
| LB | ECT | 70 |
| LB | AB | 70 |
| LB | SA | 70 |
| SB | LB | 182 |
| SB | SB | 168 |
| SB | DELIV | 168 |
| SB | TRO | 70 |
| SB | ECT | 70 |
| SB | AB | 70 |
| SB | SA | 70 |
| DELIV | LB | 182 |
| DELIV | SB | 168 |
| DELIV | DELIV | 168 |
| DELIV | TRO | 70 |
| DELIV | ECT | 70 |
| DELIV | AB | 70 |
| DELIV | SA | 70 |
| TRO | LB | 168 |
| TRO | SB | 154 |
| TRO | DELIV | 154 |
| TRO | TRO | 56 |
| TRO | ECT | 56 |
| TRO | AB | 56 |
| TRO | SA | 56 |
| ECT | LB | 168 |
| ECT | SB | 154 |
| ECT | DELIV | 154 |
| ECT | TRO | 56 |
| ECT | ECT | 56 |
| ECT | AB | 56 |
| ECT | SA | 56 |
| AB | LB | 168 |
| AB | SB | 154 |
| AB | DELIV | 154 |
| AB | TRO | 56 |
| AB | ECT | 56 |
| AB | AB | 56 |
| AB | SA | 56 |
| SA | LB | 168 |
| SA | SB | 154 |
| SA | DELIV | 154 |
| SA | TRO | 56 |
| SA | ECT | 56 |
| SA | AB | 56 |
| SA | SA | 56 |

###### 2nd Step in the Pregnancy Episode Algorithm: Assignment of Start Dates

In the pregnancy episode algorithm time periods need to be defined for various elements in order to calculate pregnancy start date. In Table 2 below the following time periods for each outcome are defined:

- Maximum pregnancy term - amount of time to search back from the outcome for all pregnancy markers. This time period should be the longest possible gestation period for an outcome so as to capture early markers.
- Minimum pregnancy term - Shortest possible gestational period for each outcome.
- Number of days a 2^nd^ pregnancy is possible after the outcome – This time window indicates how many days after the outcome specified another pregnancy may be initiated.

All time windows were estimated from research specific to each outcome and validated by in-house clinical experts.

| **Table 2: Time windows in days necessary to calculate pregnancy episode start date** | | | |
| --- | --- | --- | --- |
| **Outcome** | **Maximum Pregnancy Term** | **Minimum Pregnancy Term** | **Days 2nd pregnancy possible after outcome** |
| **LB** | 301 | 161 | 28 |
| **SB** | 301 | 140 | 28 |
| **DELIV** | 301 | 140 | 28 |
| **TRO** | 112 | 42 | 14 |
| **ECT** | 84 | 42 | 14 |
| **AB** | 168 | 42 | 14 |
| **SA** | 133 | 28 | 14 |

**Commonly administered pregnancy screening tests**

It is feasible to use common pregnancy screening tests such as Alpha fetoprotein (AFP) usually administered within narrow gestational-age windows to determine start of pregnancy episode. An assessment in Optum of the accuracy of these screening tests in determining length of pregnancy episode was conducted and of the 4 most commonly performed tests in the table below, AFP and nuchal ultrasound produced enough accuracy. However, the second algorithm in Margulis et al. [[2](#_ENREF_2)] attempted to determine beginning of pregnancy with screening test claims such as alpha fetoprotein in live births and only 45.4% of estimated gestational age at birth were within 1 week of the hospital discharge estimate; they had better success with outcome-specific estimates. For this reason, in our algorithm, we will discard any AFP or nuchal ultrasound calculated start dates that create an episode shorter or longer than the minimum and maximum allowed episode for that outcome in Table 3.

| **Table 3: Median length of pregnancy episode in days when commonly administered pregnancy screening tests are used to infer episode start date** | | | | |
| --- | --- | --- | --- | --- |
| **Pregnancy screening test** | **Clinical gestational age at which test is recommended in weeks** | **Optum Gestational age used at date of test in weeks** | **Optum median length of pregnancy in days with start date calculated from screen test** | **Comment** |
| **Alpha fetoprotein** | 15-20 | 17.5 | 276.5 | present in 1/3 live births;Q3=284 days |
| **Amniocentesis** | 15-20 | 17.5 | 258 | too few done in population |
| **Nuchal ultrasound** | 11.5-13.85 | 12.65 | 279 | Present in 16% live births;Q3=285 days |
| **Gestational Diabetes screening** | 24-28 | 26 | 272 | glucose screenings done prior to 26 week screen;Q3=386 days |

**Pregnancy Episode Start Date Calculation**

To calculate pregnancy episode start date possible start dates will be assigned in the following hierarchy (final start date chosen will be first one assigned in hierarchy):

**Step 1:** LMP (last menstrual period) date as start of pregnancy episode:

1. If LMP records exists between (larger of maximum term allowed subtracted from outcome date or prior outcome date plus retry count) and minimum term allowed subtracted from outcome date then LMP becomes the pregnancy start date
2. If there are more than one LMP records in the live birth window, use the latest so as not to grab a LMP record from an earlier pregnancy episode
3. ~50% of CPRD live births will have an LMP value (median pregnancy length 280 days using LMP date as start of episode); no US claims live births will

**Step 2:** GEST (gestational age in weeks) date as start of pregnancy episode:

1. In CPRD, for GEST record use string_as_value from observation table or in CPRD or US CLAIMS use gest_value from pregnancy concept file, whichever is present. Only use GEST values between 0 and 42 weeks.
2. If GEST record date-(GEST value*7) +1 exists between (larger of maximum term allowed subtracted from outcome date or prior outcome date plus retry count) and minimum term allowed subtracted from outcome date then GEST record date-(GEST value*7) +1 becomes the pregnancy start date.
3. If there are more than one GEST records in the allowed time window, use the latest so as not to grab a GEST record from an earlier episode
4. ~28% of CPRD live births will have a GEST value and not a LMP value with a median pregnancy length 279 days (in CPRD GEST codes are used for any outcome in the additional file); 1.4% of Optum live births will have a GEST value with a median pregnancy length 230 days (in OPTUM GEST codes are used primarily to record a premature birth)

**Step 3:** NULS (nuchal ultrasound) date as start of pregnancy episode:

1. If NULS date-89 exists between (larger of maximum term allowed subtracted from outcome date or prior outcome date plus retry count) and minimum term allowed subtracted from outcome date then NULS date-89 becomes the pregnancy start date.
2. If there are more than one NULS records in the allowed time window, use the first so as to use the NULS date that is most likely the recommended date in clinical guidelines rather than a repeat test.

**Step 4:** AFP (alpha fetoprotein test) date as start of pregnancy episode:

1. If AFP date-123 exists between (larger of maximum term allowed subtracted from outcome date or prior outcome date plus retry count) and minimum term allowed subtracted from outcome date then AFP date-123 becomes the pregnancy start date.
2. If there are more than one AFP records in the allowed time window, use the first so as to use the AFP date that is most likely the recommended date in clinical guidelines rather than a repeat test.

**Step 5:** Outcome specific estimates:

1. If at least one PREM (premature term pregnancy), POSTT (post term pregnancy) or FT (full term pregnancy) record exists between (larger of maximum term allowed subtracted from outcome date or prior outcome date plus retry count) and 30 days after outcome date then categorize pregnancy as PREM, POSTT or FT.
2. If combinations of PREM, POSTT, FT records exist in this time window, categorize pregnancy in this order: 1) PREM if present, 2) POSTT if present, 3) FT if present.
3. Based on Table 4, choose an outcome specific gestational age estimate based on outcome and term and subtract from outcome date to obtain a pregnancy start date. If prior outcome date plus retry count is greater than outcome date minus outcome specific gestational age estimate then pregnancy start date becomes prior outcome date plus retry count.

**Step 6**: Choose final pregnancy start date:

1. Choose the best pregnancy start date (first one assigned in steps 1-5).
2. If a PCONF (pregnancy confirmation), AGP (antenatal GP visit) or OTEST (other pregnancy test) category records exist between (larger of maximum term allowed subtracted from outcome date or prior outcome date plus retry count) and minimum term allowed subtracted from outcome date and is prior to the best start date as calculated in the prior 5 steps then the first found PCONF, AGP or OTEST becomes pregnancy start date.

Table 4 provides outcome specific gestational age estimates for all outcomes for premature, post and full term pregnancies. These outcome specific gestational age estimates were obtained from Hornbrook et al.[[1](#_ENREF_1)] and Margulis et al. and were validated by in-house clinical experts and should represent average gestational times for the outcomes and terms.

| **Table 4: Outcome Specific Gestational Age Estimates in Days** | | | | |
| --- | --- | --- | --- | --- |
| **Outcome** | **PreTerm** | **PostTerm** | **FullTerm** | **NoData** |
| **LB** | 245 | 285 | 280 | 280 |
| **SB** | 196 | 285 | 280 | 196 |
| **DELIV** | 245 | 285 | 280 | 280 |
| **TRO** | 56 | 56 | 56 | 56 |
| **ECT** | 56 | 56 | 56 | 56 |
| **AB** | 70 | 70 | 70 | 70 |
| **SA** | 70 | 70 | 70 | 70 |

#### Potential Confounders and Effect Modifiers

In this descriptive summary of pregnancy episodes, no confounding adjustment is performed.

### Analyses

#### Specific Comparisons

No comparisons will be made. All database results will be presented independently, with no explicit comparisons across databases performed.

### Missing Data Handling

In this descriptive summary of pregnancy episodes, we assume the existence of pregnancy and the outcome to establish the cohorts. Patients with missing year of birth are excluded before inclusion into the OMOP CDM. Any unobserved diagnoses are regarded as absence of evidence.

### Data Preparation

The primary data quality assurance activities are performed through the development of OMOP CDM, which is documented elsewhere (<http://omop.org/CDM>).

### Statistical Analysis Plan

#### Descriptive Analyses

Counts of pregnancy episodes and distribution of outcomes will be produced for this study. Also distributions of pregnancy terms by outcome will be reviewed.

#### Quantitative Analyses

This is a methodologic study, so no quantitative comparisons will be made.

#### Sensitivity and Other Supplementary Analyses

No pre-specified sensitivity analysis will be performed.

### Data Quality Assurance

#### Validation Procedures

The primary data quality assurance activities are performed through the development of OMOP CDM, which is documented elsewhere (<http://omop.org/CDM> ).

The analysis will be programmed by a member of the Janssen Global Epidemiology Analytics team. A second programmer will perform an independent quality assurance review of all source code and analysis results to confirm the work performed is consistent with what is described in this protocol before the results are shared with the investigator team.

Validation efforts will include a review of 50 CPRD and 50 Optum randomly drawn pregnancy episodes from each outcome (6 outcomes X 50 X 2) for a total of 600 profiles by clinical personnel. An extra 50 profiles for LB outcomes for each database will also be reviewed because the majority of pregnancy episodes result in a live birth. Proportion of episodes which refer to an actual pregnancy, proportion of episodes with correct outcome and proportion of episodes with correct start date (or plausible start date if inferred) for each database will be determined.

## STUDY LIMITATIONS

Truven CCAE and Medicaid and Optum databases only capture information reported through the payer, so any non-reimbursable exposures or health service utilization from other sources may not be observed in these data sources. In CPRD the general practices receive information from patient specialist visits but are not required to enter the data if it is not needed to inform future patient care. Births that do not occur in an inpatient setting most likely will not be captured in a claims database. Entire pregnancy episodes may not be captured by our algorithm, especially miscarriages occurring early in the pregnancy that require no medical care. Misclassification of outcomes could occur due to ambiguous outcome source codes.

## ETHICAL ASPECTS

### Privacy of Personal Data

Confidentiality of patient records will be maintained at all times. All analyses of electronic claims will be performed using appropriately de-identified data without access to personal identifying information. All study reports will contain aggregate data only and will not identify individual patients or physicians. Medical record abstraction will not be performed.

References

1. Hornbrook, M.C., et al., *Development of an algorithm to identify pregnancy episodes in an integrated health care delivery system.* Health Serv Res, 2007. **42**(2): p. 908-27.

2. Margulis, A.V., et al., *Algorithms to estimate the beginning of pregnancy in administrative databases.* Pharmacoepidemiol Drug Saf, 2013. **22**(1): p. 16-24.

3. Hardy, J.R., et al., *Strategies for identifying pregnancies in the automated medical records of the General Practice Research Database.* Pharmacoepidemiol Drug Saf, 2004. **13**(11): p. 749-59.

4. Devine, S., et al., *The identification of pregnancies within the general practice research database.* Pharmacoepidemiol Drug Saf, 2010. **19**(1): p. 45-50.

5. Cea-Soriano, L., et al., *Challenges of using primary care electronic medical records in the UK to study medications in pregnancy.* Pharmacoepidemiol Drug Saf, 2013. **22**(9): p. 977-85.

6. Andrade, S.E., et al., *Use of prescription medications with a potential for fetal harm among pregnant women.* Pharmacoepidemiol Drug Saf, 2006. **15**(8): p. 546-54.

7. Yang, T., et al., *Maternal characteristics associated with pregnancy exposure to FDA category C, D, and X drugs in a Canadian population.* Pharmacoepidemiol Drug Saf, 2008. **17**(3): p. 270-7.

8. Cooper, W.O., G.B. Hickson, and W.A. Ray, *Prescriptions for contraindicated category X drugs in pregnancy among women enrolled in TennCare.* Paediatr Perinat Epidemiol, 2004. **18**(2): p. 106-11.

9. Matcho, A., et al., *Fidelity assessment of a clinical practice research datalink conversion to the OMOP common data model.* Drug Saf, 2014. **37**(11): p. 945-59.

10. Rijnbeek, P.R., *Converting to a common data model: what is lost in translation? : Commentary on "fidelity assessment of a clinical practice research datalink conversion to the OMOP common data model".* Drug Saf, 2014. **37**(11): p. 893-6.

11. Mikolajczyk, R.T., A.A. Kraut, and E. Garbe, *Evaluation of pregnancy outcome records in the German Pharmacoepidemiological Research Database (GePaRD).* Pharmacoepidemiol Drug Saf, 2013. **22**(8): p. 873-80.

**APPENDIX 1: Disease and Drug Codes**
